# Synthesis of N-doped chiral macrocycles by regioselective palladium-catalyzed arylation

**DOI:** 10.3762/bjoc.21.149

**Published:** 2025-09-15

**Authors:** Shuhai Qiu, Junzhi Liu

**Affiliations:** 1 Department of Chemistry, The University of Hong Kong, Pokfulam Road, Hong Kong, China; 2 State Key Laboratory of Synthetic Chemistry, HKU-CAS Joint Laboratory on New Materials and Shanghai-Hong Kong Joint Laboratory on Chemical Synthesis, The University of Hong Kong, Pokfulam Road, Hong Kong, China; 3 Materials Innovation Institute for Life Sciences and Energy (MILES), HKU-SIRI, Shenzhen, China

**Keywords:** dihydroindolocarbazole, inherent chirality, N-doped macrocycle, nonplanarity, regioselective cyclization

## Abstract

A series of nitrogen (N)-doped macrocycles was successfully synthesized through palladium-catalyzed arylation. X-ray crystallographic characterization revealed the formation of isomeric products depending on the substituents on the N atoms. Notably, two intrinsically chiral macrocycles **MC1** and **MC3** with *C*_1_ symmetry were successfully obtained. These macrocycles exhibit exceptional photophysical properties, particularly remarkable high fluorescence quantum yields (Φ_F_ up to 0.69). Furthermore, enantiomeric resolution of inherent chiral **MC1** was achieved using preparative chiral HPLC, enabling detailed investigation of its chiroptical behavior through circular dichroism and circularly polarized luminescence spectroscopy.

## Introduction

**Figure 1:**
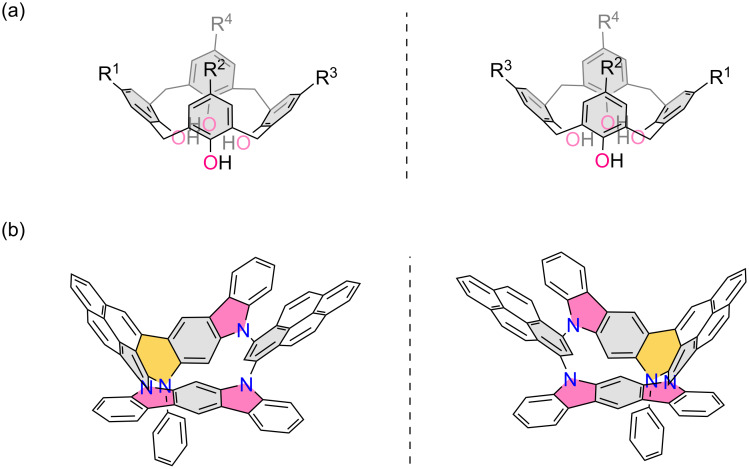
(a) Representatives of inherent chiral calix[4]arenes. (b) Molecular skeletons of inherent chiral N-doped macrocycles in this work.

Chiral macrocycles have attracted significant research interest owing to their diverse applications in enantioselective recognition [1–2], catalysis [3–4], and circularly polarized luminescence [5–6]. Generally, chirality in macrocycles arises from subunits featuring classical chiral elements [7], such as central, axis, planar and helical configurations. In contrast, inherent chirality represents a non-classical phenomenon where chirality emerges from the rigid and nonplanar architecture of macrocycles that inherently lacks symmetry [8–9]. One of the most typical representatives are calix[4]arenes (Figure 1a), first reported by Böhmer in 1994 [10], where asymmetric substitutions on the macrocyclic rim induce inherent chirality. Subsequent advancements have identified other inherent chiral systems, including molecular bowls [11–13] and medium-sized macrocycles containing a saddle-shaped eight-membered ring [14–15]. In the past decades, despite rapid progress in chiral macrocycles, inherent chirality is largely limited to calix[*n*]arene derivatives. This underscores a critical opportunity to design novel macrocyclic frameworks with intrinsic asymmetry.

Nitrogen (N)-doped macrocycles are of peculiar interest due to their unique optical, electronic and magnetic properties [16–19]. Among them, aza[1*_n_*]metacyclophanes, in which *m*-phenylene units are linked via N atoms, serves as N-bridged structural analogs of [1*_n_*]metacyclophanes. In comparison to all-carbon [1*_n_*]metacyclophanes, the incorporation of N atoms endows them with unique features, such as enhanced molecular dynamics and tunable redox property, positioning them as key precursors to construct organic high-spin materials [20–23]. In addition to benzene-based systems, pyridine-embedded aza[1*_n_*]metacyclophanes have been synthesized by Wang [24]. Despite these advances, N-doped chiral macrocycles incorporating extended π-conjugated moieties remain largely underexplored. To date, only a few examples, carbazole-based chiral macrocycles, have been reported [17,25], highlighting a critical gap in the design of chiral macrocycles with tailored electronic landscapes. Herein, we reported the synthesis, characterizations and photophysical properties of inherent chiral N-doped macrocycles (Figure 1b) via regioselective palladium (Pd)-catalyzed arylation of aza[1_4_]metacyclophane derivatives. By modulating the substitutions on the N atoms, two isomeric macrocycles, a *C*_1_-symmetric one as the minor fraction (**MC1**) and a *C*_2_*_v_*-symmetric one as the major product (**MC2**), were successfully obtained when 4-*tert*-butylphenyl groups were introduced. In contrast, when bulky 3,5-bis(trifluoromethyl)phenyl groups were introduced, only inherent chiral macrocyclic products (**MC3**) were obtained in high yield. Their molecular structures are unambiguously characterized by NMR, mass spectra and X-ray crystallographic characterization. In addition, these macrocycles show blue to green emissions with high fluorescence quantum yields (Φ_F_ up to 0.69). Owing to the existence of inherent chirality, two enantiomers of N-doped macrocycle **MC1** were successfully isolated by chiral resolution, enabling detailed investigation of its chiroptical properties through circular dichroism (CD) and circularly polarized luminescence (CPL) spectroscopy.

## Results and Discussion

**Scheme 1:**
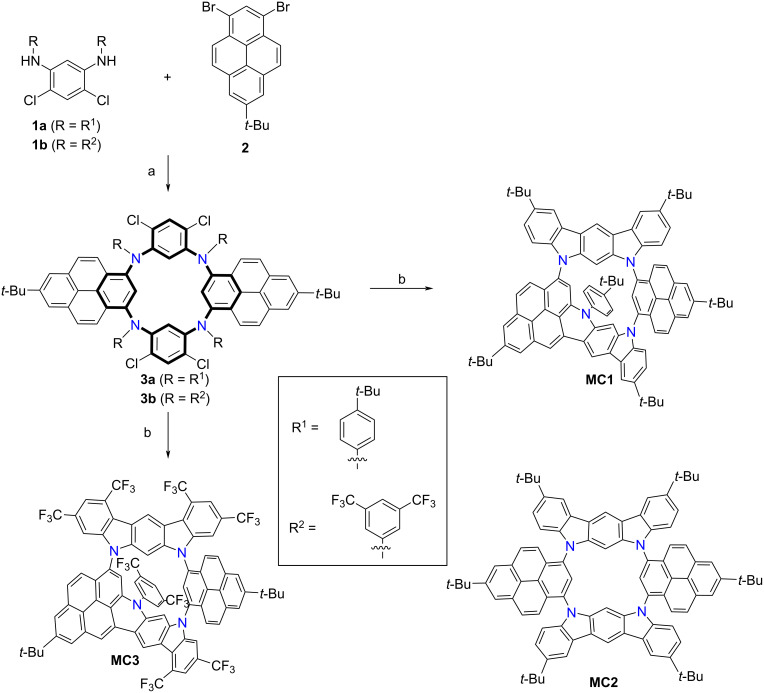
Synthesis of N-doped macrocycles **MC1**, **MC2**, and **MC3**. Reaction conditions: a) Pd_2_(dba)_3_, P*t*-Bu_3_·HBF_4_, NaO*t-*Bu, toluene, 110 °C, 24 h. **3a**: 16%; **3b**: 10%. b) Pd(OAc)_2_, PMe(*t*-Bu)_2_·HBF_4_, 1,8-diazabicyclo[5.4.0]undec-7-ene (DBU), DMAc, microwave, 170 °C, 5 h. **MC1**: 5%; **MC2**: 90%; **MC3**: 85%.

The syntheses of N-doped macrocycles **MC1**–**3** are shown in Scheme 1. Diamines **1a** and **1b** were synthesized by double Pd-catalyzed C–N coupling reaction of 4,6-dichlorobenzene-1,3-diamine with phenyl bromide (see Supporting Information File 1). Subsequent Buchwald–Hartwig reaction with 1,3-dibromo-7-*tert*-butylpyrene (**2**) gave the [2 + 2] macrocyclic precursors **3a**,**b** as the major product in 16%/10% yields, and trace amounts of higher oligomers as detected by mass spectrometry. Notably, compounds **3a**,**b** could be viewed as the aza[1_4_]metacyclophane derivatives, in which two benzene rings are replaced by two pyrenes. The Pd-catalyzed arylation of **3a** with Pd(OAc)_2_, PMe(*t*-Bu)_2_·HBF_4_ and DBU under microwave conditions gave two isomeric macrocycles **MC1** and **MC2** with four newly formed C–C bonds in yields of 5% and 90%, respectively. For **MC2,** four C–C bonds are formed between the dichlorobenzene units and *tert*-butylphenyl groups, generating two dihydroindolo[2,3-*b*]carbazole subunits. In contrast, there is only one newly formed C–C bond between the dichlorobenzene unit and one pyrene moiety for **MC1**. Interestingly, for the cyclization of **3b**, only compound **MC3** was obtained in 85% yield, which is probably attributed to larger steric hindrance deriving from bis(trifluoromethyl)phenyl groups. These macrocycles show good solubility in common solvents, and their chemical structures have been unambiguously characterized by NMR spectroscopy, mass spectrometry, and X-ray crystallography.

**Figure 2:**
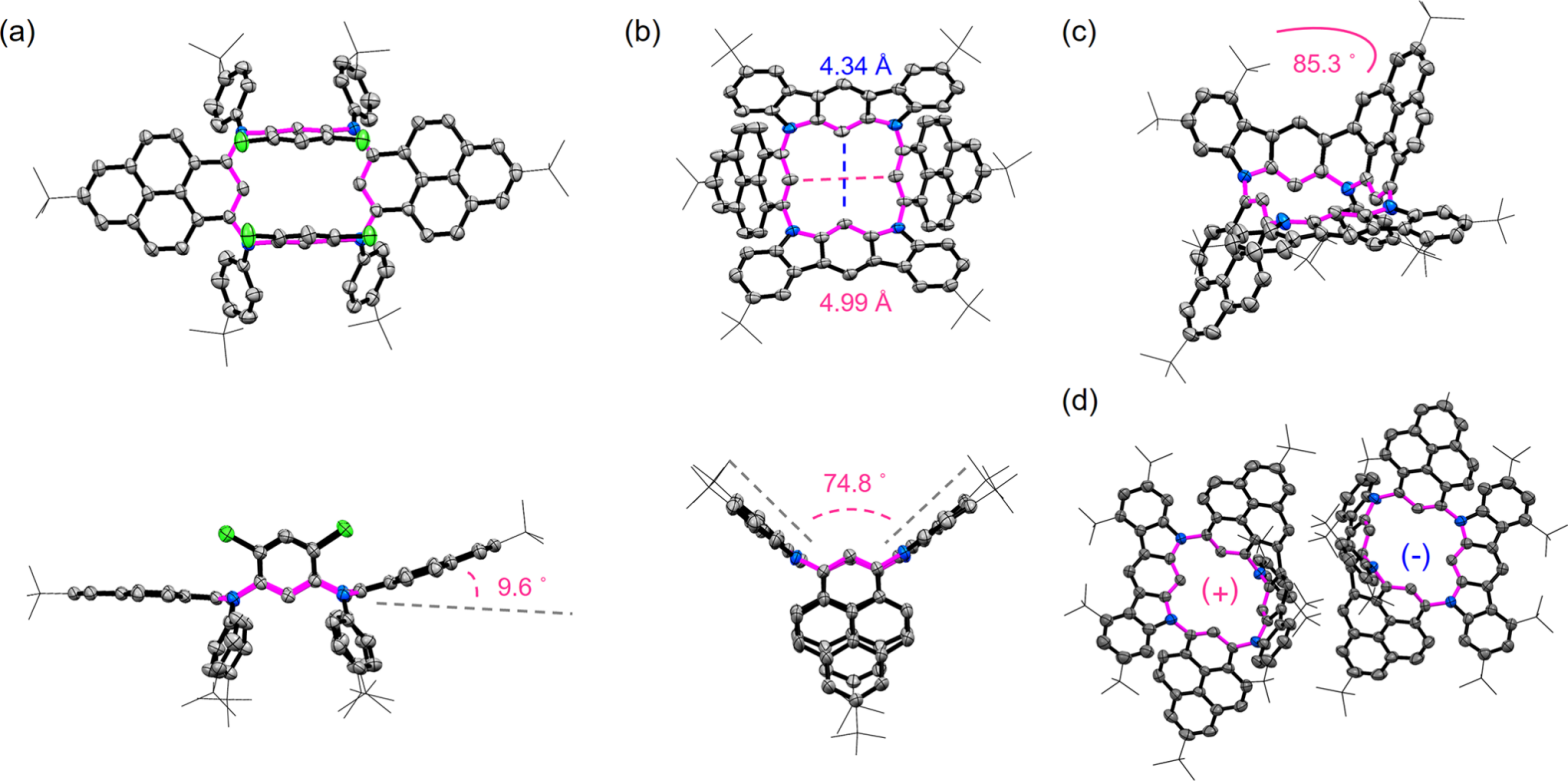
Crystal structures of compounds (a) **3a**, (b) **MC2**, and (c) **MC3**. (d) Molecular arrangements of **MC3**. Hydrogen atoms are omitted for clarity.

Single crystals suitable for X-ray diffraction measurements of compounds **3a**, **MC2**, and **MC3** were successfully obtained to reveal their molecular structures. In the crystal structure of **3a** (Figure 2a), the two pyrene units are nearly coplanar with a dihedral angle of 170°. The two dichlorobenzene rings are parallel to each other and perpendicular to the pyrene plane, and the four *tert*-butylphenyl groups are directed on one side of the pyrene plane to minimize steric repulsion. **MC2** takes a *C*_2v_-symmetric saddle-shaped geometry with two planar dihydroindolo[2,3-*b*]carbazole subunits orienting upwards with a dihedral angle of 75° and two pyrene units downwards (Figure 2b). Besides, the central cavity is highly symmetric, and the shortest diameters are determined to be 4.34 Å and 4.99 Å, respectively. In contrast to **MC2**, **MC3** shows an asymmetric geometry due to the fusion of the pyrene unit (Figure 2c). The two pyrene units are oriented antiparallel, which is distinctive from that observed in **3a** and **MC2**. Notably, the pyrene-fused moiety is highly curved with a bending angle of 85.3° as defined by the angle of the planes of the terminal rings. In the molecular arrangement, a pair of enantiomers exists in each cell for **MC3** (Figure 2d). Considering the C–C single bonds between the π-subunits, isomerization among different molecular configurations might occur via rotations. To further investigate the conformational stability of **MC3**, theoretical calculations were performed to evaluate the energy barriers of isomerization. As shown in Figure S3 (Supporting Information File 1), the configuration observed in the crystal structure has the lower energy by 24.0 kcal mol^−1^ than that of the isomeric structure with two pyrene units at the same side. The energy barrier was calculated to be 66.7 kcal mol^−1^, indicating **MC3** is highly conformationally stable.

**Figure 3:**
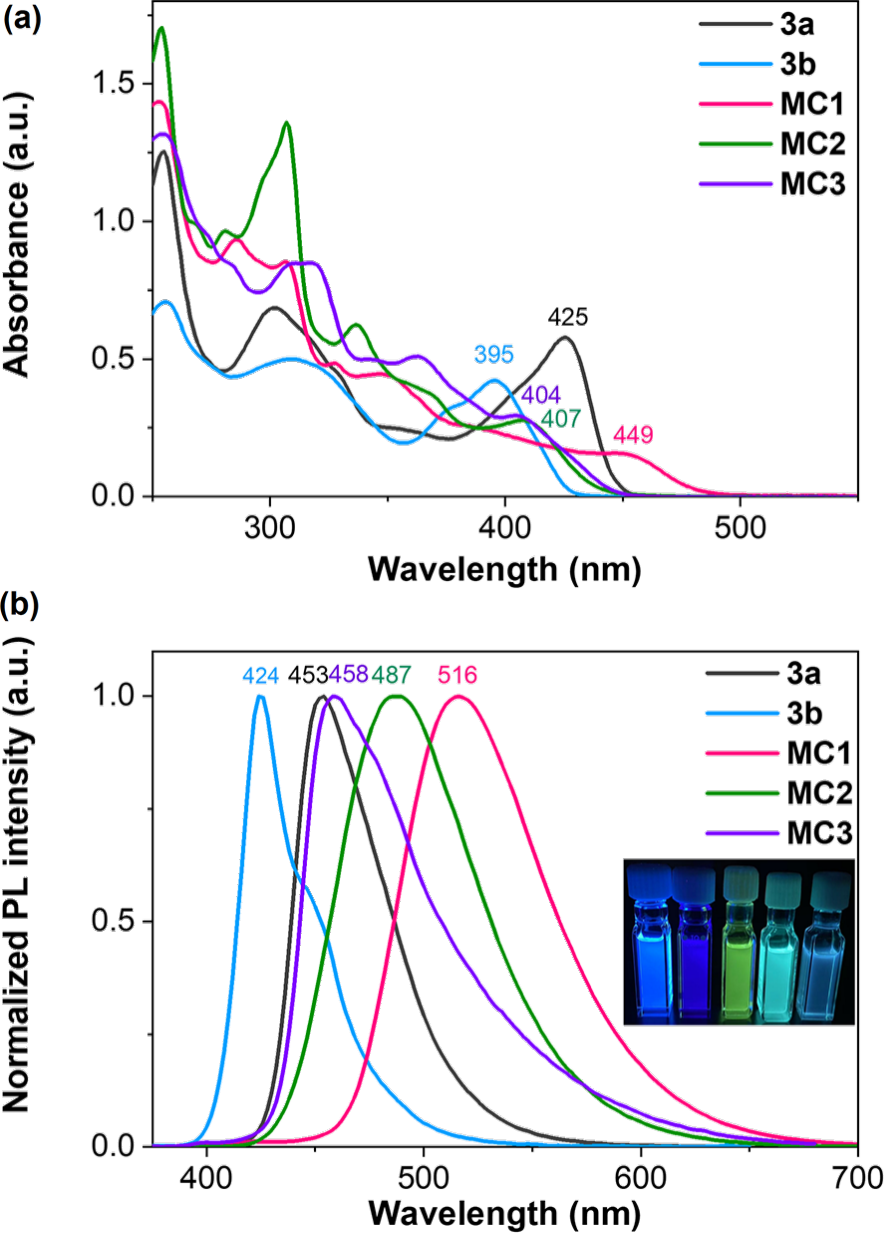
(a) Absorptions and (b) emissions of compounds **3a**, **3b**, **MC1**, **MC2**, and **MC3** measured in dichloromethane at room temperature. The inset shows the photographs under UV light at 365 nm. The concentration is 10 μM.

The optical properties of the synthesized macrocycles were investigated in dichloromethane (Figure 3). The precursors **3a**,**b** show intense absorptions with maxima at 425 nm and 395 nm, respectively. Correspondingly, **3a** exhibits a deep blue emission at 453 nm with a quantum yield (Φ_F_) of 0.79, while a hypsochromic shift of the signal for **3b** to 424 nm is observed and the Φ_F_ value is decreased to 0.22 due to the electron-deficient character of the 3,5-bis(trifluoromethyl)phenyl groups. The absorption maximum of **MC1** is more redshifted by 42 nm compared to **MC2**, which is attributed to the extended conjugation after the fusion of one pyrene unit. Similarly, both **MC1** and **MC2** have higher Φ_F_ values of 0.45 and 0.69 than compound **MC3** (Φ_F_ = 0.13). The optical energy bandgaps were determined to be 2.48 eV for **MC1**, 2.61 eV for **MC2**, and 2.68 eV for **MC3**, respectively, based on the onset absorptions. **MC2** and **MC3** display strong blue emissions at 487 nm and 458 nm, respectively, while **MC1** exhibits green photoluminescence at 516 nm.

**Figure 4:**
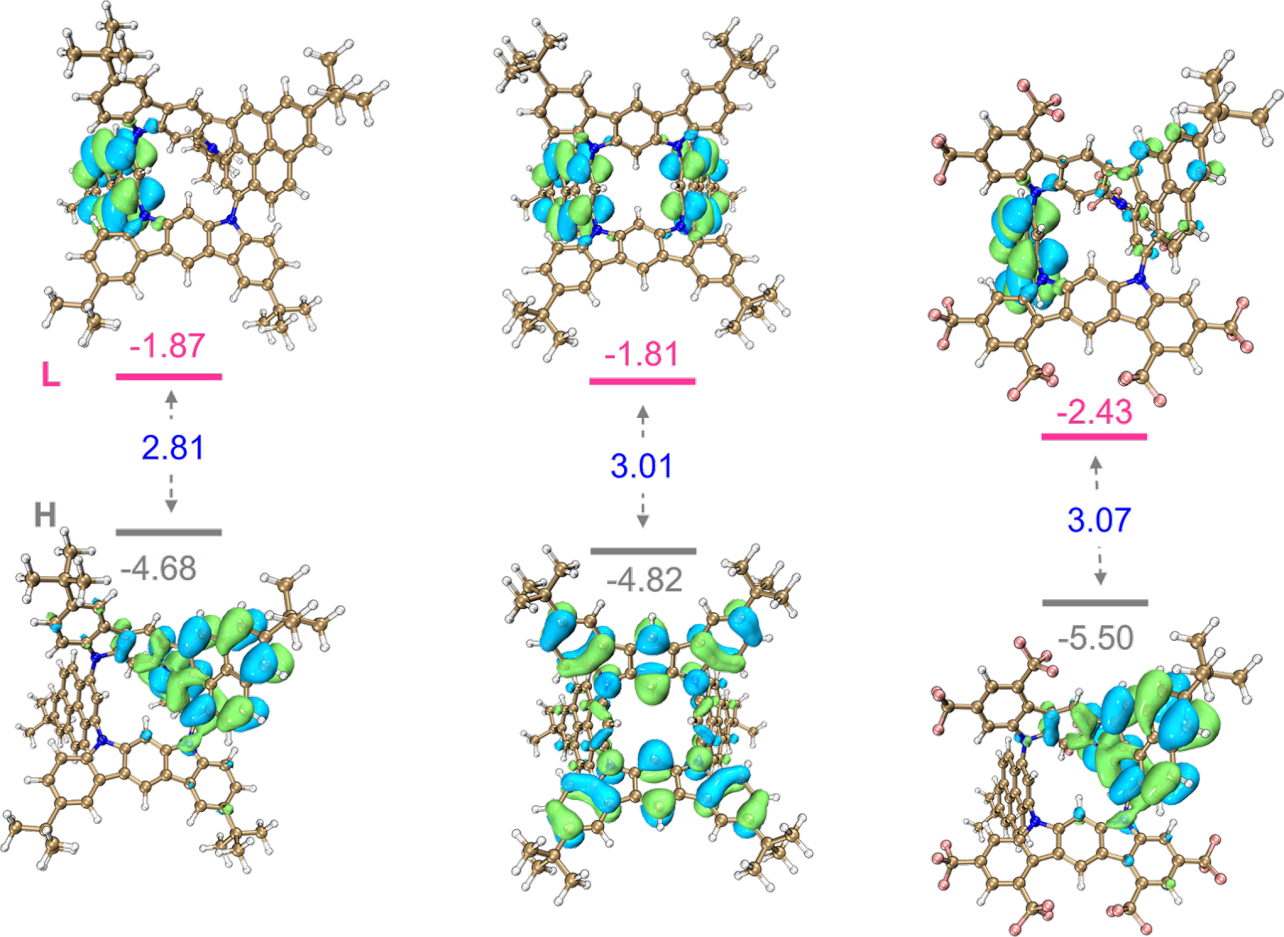
Calculated frontier molecular orbitals and relative energy levels of **MC1** (left), **MC2** (middle), and **MC3** (right) calculated at the B3LYP/6-31G(d) level of theory.

To better understand the electronic structures of these N-doped macrocycles, theoretical calculations on the frontier molecular orbitals were carried out based on the optimized structures. As shown in Figure 4, the distributions of the highest occupied molecular orbitals (HOMOs) are disjointed from that of the lowest unoccupied molecular orbitals (LUMOs). Specifically, the HOMOs of **MC1** and **MC3** mainly distribute on the fused pyrene moiety and the substituent on the N atom, while the LUMOs localize on the other pyrene unit. In contrast, the HOMOs of **MC2** are mainly located on two dihydroindolo[2,3-*b*]carbazole subunits, and the LUMOs localize on two pyrene units. Owing to electron-deficient character, both the HOMO and LUMO energy levels of **MC3** are obviously decreased in comparison to **MC1** and **MC2**. Accordingly, the calculated energy gaps are 2.81 eV for **MC1**, 3.01 for **MC2** and 3.08 for **MC3**, respectively, which are in line with the optical ones.

**Figure 5:**
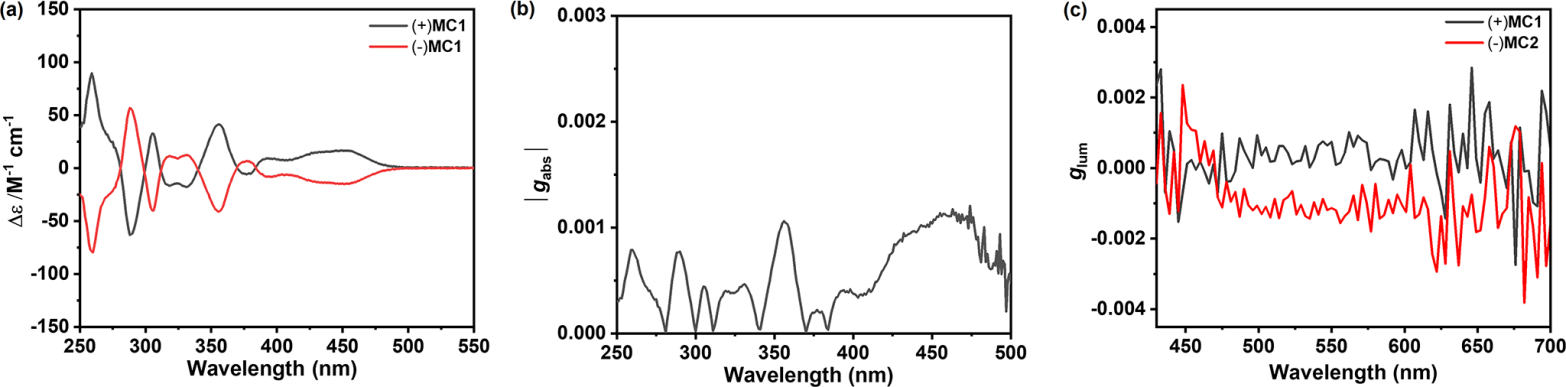
(a) CD spectra, (b) |*g*_abs_|, and (c) *g*_lum_ values of enantiomers of **MC1** measured in dichloromethane at room temperature. The concentrations were 10 μM.

In view of the existence of inherent chirality for both **MC1** and **MC3**, chiral resolutions using chiral high-performance liquid chromatography (HPLC) were performed. Due to insufficient solubility, **MC3** failed in chiral separation via preparative chiral columns. Fortunately, two enantiomers of **MC1** were successfully isolated with a Daicel Chiralpak IF column (Figure S1, Supporting Information File 1). The absolute configuration of the separated enantiomers of **MC1** was determined based on the calculated CD spectra (Figure S4, Supporting Information File 1). The first fraction was defined as the (+)-enantiomer, and the second fraction was assigned as the (−)-enantiomer. As shown in Figure 5, the CD spectra displayed mirror images with positive and negative Cotton effects at wavelengths from 250 to 500 nm, indicating strong chiroptical responses. (+)-**MC1** shows five positive Cotton effects at 259, 305, 355, 392, and 453 nm, as well as four negative Cotton effects at 288, 317, 331, and 432 nm, respectively. (−)-**MC1** exhibits a mirror image with the opposite signals to that of (+)-**MC1**. The maximum absorption dissymmetry factor (*g*_abs_) value of 1.1 × 10^−3^ at 453 nm is observed (Figure 5b), which is derived from the S_0_→S_1_ transition. Similar to the CD spectra, mirror images of the CPL spectra (Figure S2, Supporting Information File 1) and luminescence dissymmetry factor (*g*_lum_) plots (Figure 5c) were observed for the enantiomers of **MC1**. However, both enantiomers show a low *g*_lum_ value below 1.0 × 10^−3^.

## Conclusion

In summary, we demonstrated the synthesis and characterizations of N-doped macrocycles **MC1**–**3** by palladium-catalyzed arylations. The molecular structures of the macrocyclic precursors and targets were unambiguously revealed by X-ray crystallographic characterization. These macrocycles exhibit strong fluorescence with Φ_F_ values up to 0.69. Remarkably, **MC1** and **MC3** are inherent chiral owing to their *C*_1_ symmetric structures. The enantiomers of **MC1** were successfully isolated by chiral resolution, which indicate a *g*_abs_ value of 1.1 × 10^−3^ and a *g*_lum_ value at the level of 10^−4^. Our work represents one of the rare examples of non-classical chiral macrocycles, providing insights into molecular design of chiral macrocycles with high emissions.

## Supporting Information

File 1 Experimental procedures, synthetic details, and X-ray crystallographic data.

File 2 Crystallographic information files for compounds **3a**, **MC2**, and **MC3**.

## Data Availability

All data that supports the findings of this study is available in the published article and/or the supporting information of this article.
