# Supplementary material for: Synthesis of N-doped chiral macrocycles by regioselective palladium-catalyzed arylation
Source: Beilstein J Org Chem. 2025 Sep 15;21:1917–23. doi: 10.3762/bjoc.21.149 (PMC12456077; doi:10.3762/bjoc.21.149)
Supplement: File 1 — Experimental procedures, synthetic details, and X-ray crystallographic data. [file Beilstein_J_Org_Chem-21-1917-s001.pdf]

BEILSTEIN JOURNAL OF ORGANIC CHEMISTRY

Supporting Information

for

Synthesis of N-doped chiral macrocycles by regioselective palladium-catalyzed arylation

Shuhai Qiu and Junzhi Liu

Beilstein J. Org. Chem. **2025,** *21,* 1917–1923. doi:10.3762/bjoc.21.149

Experimental procedures, synthetic details, and X-ray crystallographic data

License and Terms: This is a supporting information file under the terms of the Creative Commons Attribution License (https://creativecommons.org/licenses/by/4.0). Please note that the reuse, redistribution and reproduction in particular requires that the author(s) and source are credited and that individual graphics may be subject to special legal provisions.
The license is subject to the *Beilstein Journal of Organic Chemistry* terms and conditions: (https://www.beilstein-journals.org/bjoc/terms)

Table of contents

1. Materials and measurements S2
2. Synthetic details S3
3. Chiral HPLC chromatograms S7
4. X-ray crystal structures S9
5. Theoretical calculations S15
6. ^{1}H and ^{13}C NMR spectra S16
7. Mass spectra S22
8. References S25

1. Materials and measurements

Solvents were purified and dried by standard methods prior to use. All commercially available reagents were used without further purification unless otherwise noted. Column chromatography was generally performed on silica gel (200–300 mesh) and reactions were monitored by thin-layer chromatography (TLC) using silica gel GF254 plates with UV light to visualize the course of reaction. The ^{1}H and ^{13}C 2D NMR data were recorded on a 500 MHz or 600 MHz spectrometer using DMSO-d_6 or CD_2Cl_2 as solvent. All chemical shifts are quoted in ppm, relative to tetramethylsilane, using residual solvent peak as a reference standard. The following abbreviations were used to explain the multiplicities: s = singlet, d = doublet, m = multiplet. Mass spectra were obtained on a Bruker Q-Tof Maxis II mass spectrometer and MALDI-TOF MS system, Bruker ultrafleXtreme. UV–vis spectroscopy was performed on an Agilent Cary 60. The photoluminescence spectra were conducted on a Cary Eclipse Fluoroscence Spectrofluorometer, Agilent Cary G9800AA. The quantum yields were measured on a Hamamatsu UV-NIR absolute PL quantum yield Spectrometer C13534. Single crystal data collections were performed on a Bruker D8 Venture with a CuKα (λ = 1.5406 Å) or GaKα (λ = 1.34139) X-ray source. All calculations were performed using the SHELXL and the crystal structure crystallographic software package.

2. Synthetic details

Synthesis of 1a

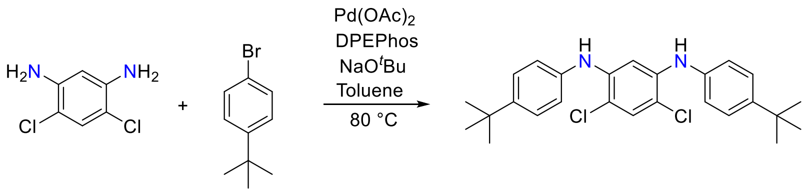

A Schlenk flask was charged with 4,6-dichlorobenzene-1,3-diamine (1.0 g, 5.7 mmol, 1 equiv), 1-bromo-4-(*tert*-butyl)benzene (2.6 g, 12.4 mmol, 2.2 equiv), $Pd(OAc)_2$ (127 mg, 10 mol %), bis[(2-diphenylphosphino)phenyl] ether (DPEphos, 456 mg, 15 mol %), NaO*t*-Bu (1.63 g, 17.1 mmol, 3 equiv) and dry toluene (50 mL) under argon. The mixture was heated to 80 °C with vigorous stirring for 15 h. The mixture was cooled and filtered, and the filtrate was evaporated to dryness. The residue was purified by the column chromatography on silica gel (petroleum ether/CH_2Cl_2 2:1) to afford **1a** as a white solid (2.5 g, 99% yield). ^{1}H NMR (600 MHz, DMSO-d_6): δ 7.49 (s, 2H), 7.39 (s, 1H), 7.29 - 7.23 (m, 4H), 7.04 (d, J =8.6 Hz, 4H), 6.92 (s, 1H), 1.22 (s, 18H). ^{13}C NMR (150 MHz, DMSO-d_6): δ 144.0, 140.5, 139.2, 129.7, 125.6, 119.8, 111.3, 103.4, 33.8, 31.2. MS (MALDI-TOF, 100%): m/z calcd (%) for $C_{26}H_{30}Cl_2N_2$: 440.1781, found: 440.1910.

Synthesis of 1b

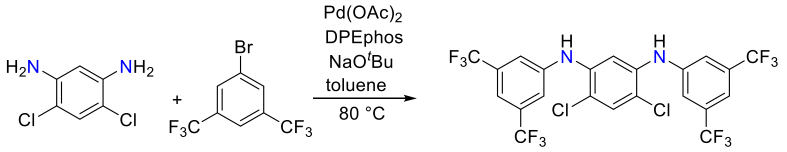

A Schlenk flask was charged with 4,6-dichlorobenzene-1,3-diamine (1.0 g, 5.7 mmol, 1 equiv), 1-bromo-4-(*tert*-butyl)benzene (3.6 g, 12.4 mmol, 2.2 equiv), $Pd(OAc)_2$ (127 mg, 10 mol %), DPEphos (456 mg, 15 mol %), NaO*t*-Bu (1.63 g, 17.1 mmol, 3 equiv) and dry toluene (50 mL) under argon. The mixture was heated to 80 °C with vigorous stirring for 15 h. The mixture was cooled and filtered, and the filtrate was evaporated to dryness. The residue was purified by column chromatography on silica gel (petroleum ether/CH_2Cl_2 2:1) to afford **1b** as a white solid (0.6 g, 19% yield). ^{1}H NMR (600 MHz, DMSO-d_6): δ 8.69 (s, 2H), 7.80 (s, 1H), 7.40 (d, J =13.3 Hz, 6H), 7.32 (s, 1H). ^{19}F NMR (565 MHz, DMSO-d_6): δ −61.9. ^{13}C NMR (150 MHz, DMSO-d_6): δ 145.7, 137.8, 131.5, 131.3, 131.3, 131.0, 130.8, 126.0, 124.2, 122.4, 121.7, 120.6, 116.6, 115.8, 115.8, 115.7, 112.0, 111.9, 111.9. MS (MALDI-TOF, 100%): m/z calcd (%) for $C_{22}H_{10}Cl_2F_{12}N_2$: 600.0024, found: 600.0613.

Synthesis of 3a

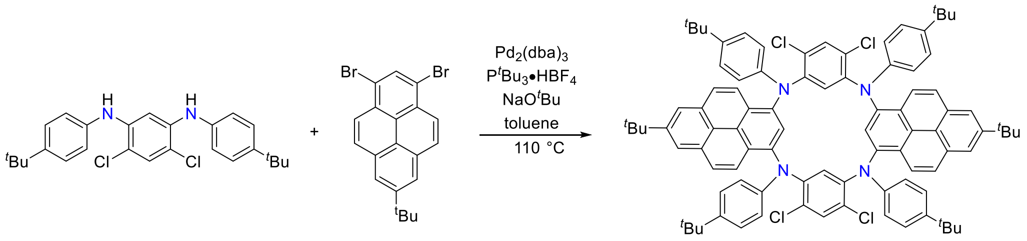

To a mixture of **1a** (218 mg, 0.5 mmol, 1 equiv), 1,3-dibromo-7-(*tert*-butyl)pyrene (208 mg, 0.5 mmol, 1 equiv), $Pd_2(dba)_3$ (23 mg, 5 mol %), tri-*tert*-butylphosphonium tetrafluoroborate (Pt-$Bu_3 \cdot HBF_4$, 29 mg, 20 mol %) and NaO*t*-Bu (144 mg, 1.5 mmol, 3 equiv), dry toluene (20 mL) was added under N_2 atmosphere. The mixture was heated at 110 °C for 24 h. Upon completion, the solution was filtrated and the filtrate was evaporated to dryness. The residue was purified by column chromatography on silica gel (petroleum ether/CH_2Cl_2 3:1) to afford **3a** (110 mg) as a yellow solid in 16% yield. It was directly utilized in the next step. **3a** shows complicated broad peaks in the ^{1}H NMR spectrum likely owing to the existence of atropisomers. MS (MALDI-TOF, 100%): m/z calcd (%) for $C_{92}H_{88}Cl_4N_4$: 1390.5755, found: 1390.5889.

Synthesis of 3b

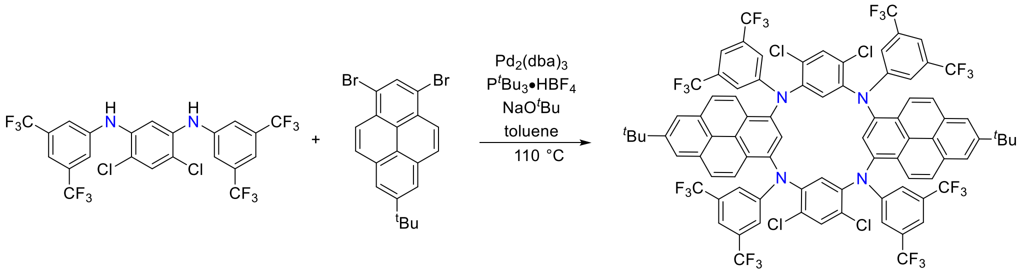

To a mixture of **1b** (120 mg, 0.2 mmol, 1 equiv), 1,3-dibromo-7-(*tert*-butyl)pyrene (84 mg, 0.2 mmol, 1 equiv), $Pd_2(dba)_3$ (7 mg, 4 mol %), Pt-$Bu_3 \cdot HBF_4$ (9 mg, 16 mol %) and NaO*t*-Bu (58 mg, 0.6 mmol, 3 equiv), dry toluene (20 mL) was added under N_2 atmosphere. The mixture was heated at 110 °C for 24 h. Upon completion, the solution was filtrated and the filtrate was evaporated to dryness. The residue was purified by the column chromatography on silica gel (petroleum ether/CH_2Cl_2 3:1) to afford **3a** (34 mg) as a white solid in 10% yield. It was directly utilized in the next step. **3b** shows complicated broad peaks in the ^{1}H NMR spectrum likely owing to the existence of atropisomers. MS (MALDI-TOF, 100%): m/z calcd (%) for $C_{84}H_{48}Cl_4F_{24}N_4$: 1710.2239, found: 1710.2702.

Synthesis of MC1 and MC2

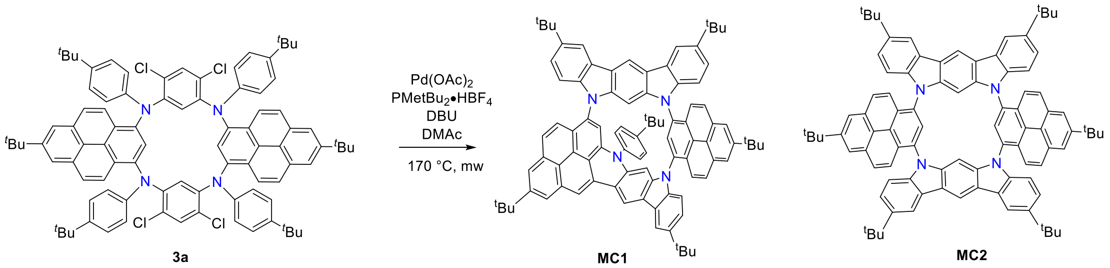

A 30 mL tube was charged with **3a** (50 mg, 0.04 mmol, 1 equiv), Pd(OAc)$_2$ (27 mg, 3 equiv), di-*tert*-butyl(methyl)phosphonium tetrafluoroborate (PMe(*t*-Bu)$_2$·HBF$_4$, 92 mg, 9 equiv), 1,8-diazabicyclo[5.4.0]undec-7-ene (DBU, 0.3 mL) and dry dimethylacetamide (5 mL) under argon. The mixture was sealed and heated to 170 °C under microwave conditions for 5 h. The mixture was cooled and poured into water. The precipitate was collected and purified by the column chromatography on silica gel (petroleum ether/CH$_2$Cl$_2$ 3:1) to afford **MC1** (2 mg, 5%) and **MC2** (40 mg, 90%) as yellow solids.

MC1: ^{1}H NMR (600 MHz, CD$_2$Cl$_2$): δ 8.87 (s, 1H), 8.80 (d, *J* =9.1 Hz, 1H), 8.40 (s, 2H), 8.38 – 8.31 (m, 3H), 8.26 (d, *J* =7.8 Hz, 2H), 8.16 (s, 1H), 8.12 (s, 1H), 8.10 (s, 1H), 8.03 (s, 1H), 7.83 (d, *J* =9.3 Hz, 1H), 7.76 (d, *J* =8.3 Hz, 1H), 7.72 – 7.66 (m, 3H), 7.48 – 7.45 (m, 1H), 7.38 (d, *J* =8.6 Hz, 1H), 7.18 (dd, *J* =8.7, 1.9 Hz, 1H), 7.07 (s, 1H), 7.04 (d, *J* =8.9 Hz, 2H), 6.87 (d, *J* =8.9 Hz, 2H), 6.72 (s, 1H), 6.64 (s, 1H), 6.55 (d, *J* =9.2 Hz, 1H), 6.46 (d, *J* =8.6 Hz, 1H), 1.59 (d, *J* =2.8 Hz, 19H), 1.53 (s, 9H), 1.43 (s, 9H), 1.39 (s, 9H), 0.98 (s, 9H). ^{13}C NMR (150 MHz, CD$_2$Cl$_2$): δ 151.1, 151.0, 150.0, 145.9, 145.5, 145.0, 144.4, 144.0, 143.9, 143.9, 143.8, 143.8, 142.4, 140.3, 139.6, 136.9, 134.6, 133.8, 133.6, 132.0, 131.7, 131.7, 131.3, 131.1, 130.6, 130.3, 129.6, 129.4, 127.2, 127.2, 127.2, 127.0, 126.9, 126.8, 126.5, 126.4, 125.7, 124.9, 124.7, 124.4, 124.2, 123.9, 123.8, 123.6, 123.5, 123.3, 122.8, 122.7, 122.7, 122.7, 121.2, 120.9, 120.7, 117.4, 117.2, 116.8, 116.3, 116.1, 112.8, 112.2, 112.1, 111.6, 111.2, 91.3, 35.8, 35.8, 35.4, 35.3, 35.2, 34.3, 32.4, 32.3, 32.2, 32.1, 32.1, 31.5. MS (MALDI-TOF, 100%): m/z calcd (%) for $C_{92}H_{84}N_4$: 1245.6723, found: 1245.6885.

MC2: ^{1}H NMR (600 MHz, CD$_2$Cl$_2$): δ 9.05 (d, *J* =3.5 Hz, 2H), 8.42 (d, *J* =3.5 Hz, 6H), 8.25 (s, 4H), 8.01 (d, *J* =9.4 Hz, 4H), 7.81 (dd, *J* =9.4, 3.0 Hz, 4H), 7.41 (dt, *J* =8.6, 2.3 Hz, 4H), 7.39 – 7.37 (m, 2H), 6.99 (dd, *J* =8.5, 3.2 Hz, 4H), 1.52 (d, *J* =3.1 Hz, 54H). ^{13}C NMR (150 MHz, CD$_2$Cl$_2$): δ 150.9, 143.8, 143.6, 141.2, 132.5, 131.4, 129.5, 128.6, 127.6, 126.9, 124.3, 124.2, 123.5, 123.3, 123.1, 119.9, 116.7, 111.8, 110.2, 89.9, 35.7, 35.3, 32.4, 32.0. MS (MALDI-TOF, 100%): m/z calcd (%) for $C_{92}H_{84}N_4$: 1245.6723, found: 1245.7137.

Synthesis of MC3

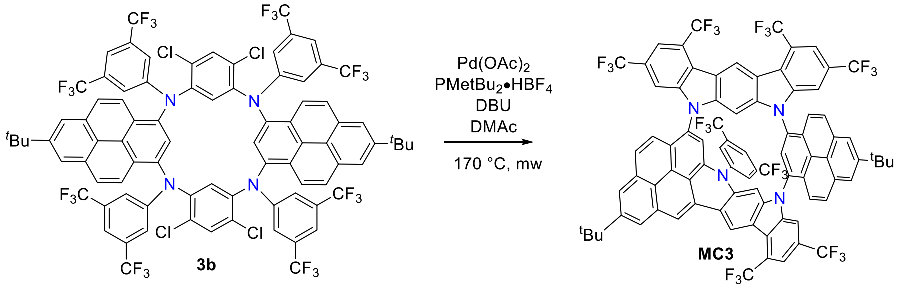

A 30 mL tube was charged with **3a** (40 mg, 0.02 mmol, 1 equiv), $Pd(OAc)_2$ (22 mg, 3 equiv), PMe(*t*-Bu)$_2$·HBF_4 (74 mg, 9 equiv), 1,8-diazabicyclo[5.4.0]undec-7-ene (DBU, 0.2 mL) and dry dimethylacetamide (5 mL) under argon. The mixture was sealed and heated to 170 °C under microwave conditions for 5 h. The mixture was cooled and poured into water. The precipitate was collected and purified by the column chromatography on silica gel (petroleum ether/CH_2Cl_2 3:1) to afford **MC3** (31 mg, 85%) as a yellow solid. ^{1}H NMR (500 MHz, CD_2Cl_2): δ 9.65 (s, 1H), 8.74 (d, *J* =9.1 Hz, 1H), 8.64 (s, 1H), 8.56 – 8.49 (m, 3H), 8.47 (s, 1H), 8.41 (s, 1H), 8.38 (s, 1H), 8.35 (s, 1H), 8.23 (d, *J* =1.7 Hz, 1H), 8.11 (s, 2H), 8.00 – 7.88 (m, 4H), 7.46 (s, 2H), 7.40 (d, *J* =9.3 Hz, 1H), 7.29 (s, 1H), 7.21 (s, 1H), 7.16 (s, 1H), 6.89 (s, 1H), 6.83 (s, 1H), 6.49 (d, *J* =9.3 Hz, 1H), 1.61 (s, 9H), 1.59 (s, 9H). ^{19}F NMR (471 MHz, CD_2Cl_2): δ -61.63, -61.81, -61.82, -62.15, -63.15, -63.17, -63.42. 13C NMR (125 MHz, CD2Cl2): δ 152.3, 152.0, 149.4, 149.0, 146.9, 145.8, 145.7, 145.4, 144.5, 142.8, 136.4, 133.5, 133.0, 132.9, 132.7, 132.6, 132.5, 132.1, 131.6, 131.5, 131.4, 131.1, 130.5, 130.4, 130.1, 129.8, 129.7, 129.4, 129.1, 127.9, 127.4, 126.6, 126.3, 126.3, 125.8, 125.6, 125.4, 125.0, 124.9, 123.8, 123.3, 123.0, 122.9, 122.1, 122.1, 121.8, 121.4, 120.7, 118.2, 118.0, 115.8, 114.7, 114.0, 113.8, 113.2, 113.0, 91.0, 36.0, 35.9, 32.1, 32.0. MS (MALDI-TOF): $[M]^+$ calcd for $C_{84}H_{44}F_{24}N_4$: 1564.3177, found 1564.3481.

3. Chiral HPLC chromatograms

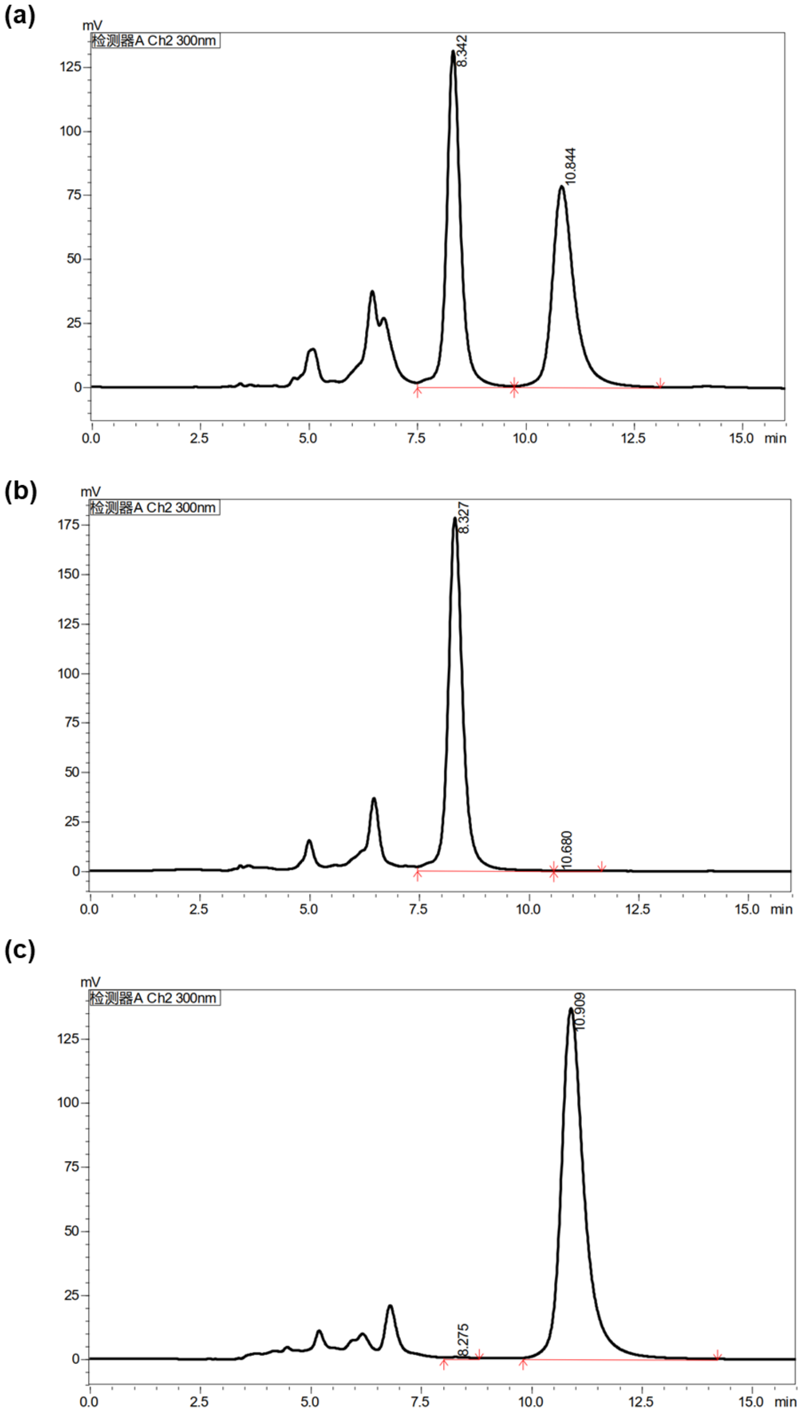

Figure S1: Resolution of **MC1** by chiral HPLC monitored at 300 nm was performed with a CHIRALPAK IF (0.46 cm I.D. × 25 cm L). Injection volume was 100 µL, and a mixture of MeOH/DCM 70:30 (V/V) was used as the eluent with a flow rate of 1.0 mL/min at 35 °C.

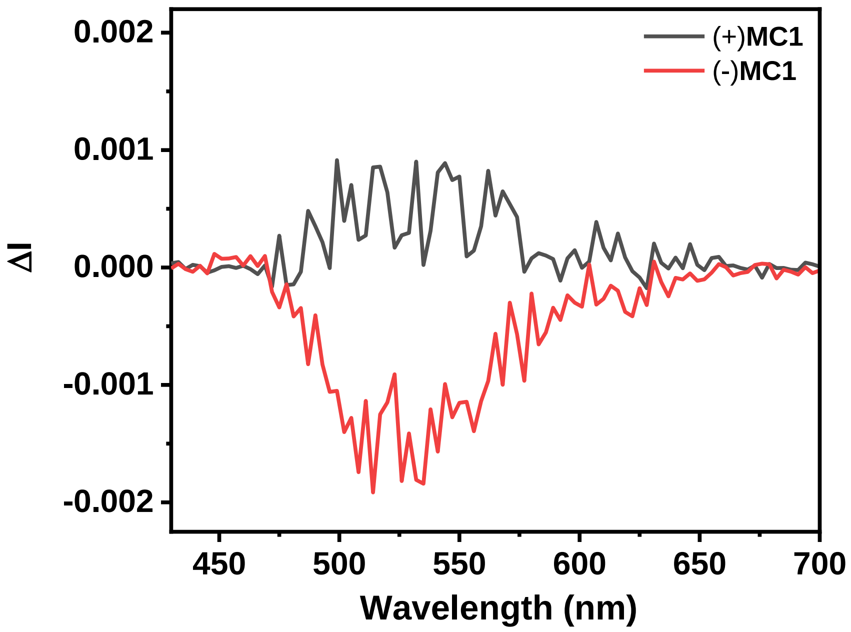

Figure S2: CPL emission spectra of enantiomers of **MC1** measured in dichloromethane at room temperature. The concentrations were 10 µM

4. X-ray crystal structures

Crystallographic data have been deposited with the Cambridge Crystallographic Data Centre as supplementary publication on No. CCDC 2451256 (**MC3**), 2451257 (**MC2**) and 2451258 (**3a**). These data are provided free of charge by the joint Cambridge Crystallographic Data Centre via https://www.ccdc.cam.ac.uk/structures/. The crystallographic data were summarized below.

Table S1. Crystal data and structure refinement for **3a**.

Identification code	3a
Empirical formula	$C_{92}H_{88}Cl_4N_4$
Formula weight	1391.46
Temperature/K	223.00
Crystal system	triclinic
Space group	P-1
a/Å	19.8177(5)
b/Å	20.7758(5)
c/Å	26.2065(6)
α /°	94.329(2)
β /°	102.462(2)
γ /°	116.5530(10)
Volume/Å^3	9240.0(4)
Z	4
ρ_{calc}g/cm^3	1.000
μ /mm^{-1}	1.471
F(000)	2944.0
Crystal size/mm^3	0.13 × 0.12 × 0.11

Radiation	CuKα (λ = 1.54178)
2Θ range for data collection/°	4.85 to 137.12
Index ranges	$-23 \leqslant h \leqslant 23$, $-22 \leqslant k \leqslant 25$, $-31 \leqslant l \leqslant 30$
Reflections collected	116982
Independent reflections	33887 [R_{int} = 0.0659, R_{sigma} = 0.0490]
Data/restraints/parameters	33887/219/1928
Goodness-of-fit on F^2	1.026
Final R indexes [I>=2σ (I)]	R_1 = 0.0664, wR_2 = 0.1953
Final R indexes [all data]	R_1 = 0.0948, wR_2 = 0.2174
Largest diff. peak/hole / e $Å^{-3}$	0.36/-0.51

Table S2. Crystal data and structure refinement for **MC2**.

Identification code	MC2
Empirical formula	$C_{92}H_{84}N_4$
Formula weight	1245.63
Temperature/K	223.00
Crystal system	triclinic
Space group	P-1
a/Å	14.6091(3)
b/Å	15.3776(4)
c/Å	22.3624(5)
α/°	92.706(2)
β/°	102.868(2)
γ/°	107.854(2)
Volume/Å^3	4625.50(19)
Z	2
ρ_{calc}g/cm^3	0.894
μ/mm^{-1}	0.390
F(000)	1328.0
Crystal size/mm^3	0.13 × 0.12 × 0.11
Radiation	CuKα (λ = 1.54178)
2Θ range for data collection/°	4.084 to 133.186
Index ranges	-13 ≤ h ≤ 17, -18 ≤ k ≤ 18, -26 ≤ l ≤ 26

Reflections collected	58057
Independent reflections	16257 [R_{int} = 0.0761, R_{sigma} = 0.0742]
Data/restraints/parameters	16257/72/914
Goodness-of-fit on F^2	1.001
Final R indexes [I>=2 σ (I)]	R_1 = 0.1085, wR_2 = 0.2607
Final R indexes [all data]	R_1 = 0.1366, wR_2 = 0.2761
Largest diff. peak/hole / e Å^{-3}	0.70/-0.39

Table S3. Crystal data and structure refinement for **MC3.**

Identification code	MC3
Empirical formula	$C_{84}H_{44}F_{24}N_4$
Formula weight	1565.23
Temperature/K	223.00
Crystal system	triclinic
Space group	P-1
a/Å	14.7541(12)
b/Å	16.6391(12)
c/Å	17.6524(14)
α /°	66.335(5)
β /°	80.415(5)
γ /°	73.726(5)
Volume/Å^3	3802.9(5)
Z	2
ρ_{calc}g/cm^3	1.367
μ /mm^{-1}	1.064
F(000)	1584.0
Crystal size/mm^3	0.13 × 0.12 × 0.11
Radiation	CuKα (λ = 1.54178)
2Θ range for data collection/°	5.476 to 137.284
Index ranges	-17 ≤ h ≤ 14, -20 ≤ k ≤ 20, -21 ≤ l ≤ 21

Reflections collected	37149
Independent reflections	13633 [R_{int} = 0.0629, R_{sigma} = 0.0569]
Data/restraints/parameters	13633/914/1199
Goodness-of-fit on F^2	1.037
Final R indexes [I>=2 σ (I)]	R_1 = 0.0748, wR_2 = 0.1716
Final R indexes [all data]	R_1 = 0.1092, wR_2 = 0.1910
Largest diff. peak/hole / e $Å^{-3}$	0.48/-0.38

5. Theoretical calculations

For all titled molecules, DFT and time-dependent DFT calculations were performed using the Gaussian 16 software package.[S1] The geometries were optimized with the B3LYP functional and 6-31G(d) basis set. Frontier molecular orbitals were analyzed with Multiwfn 3.8,[S2] and rendered with VMD 1.9.3.[S3]

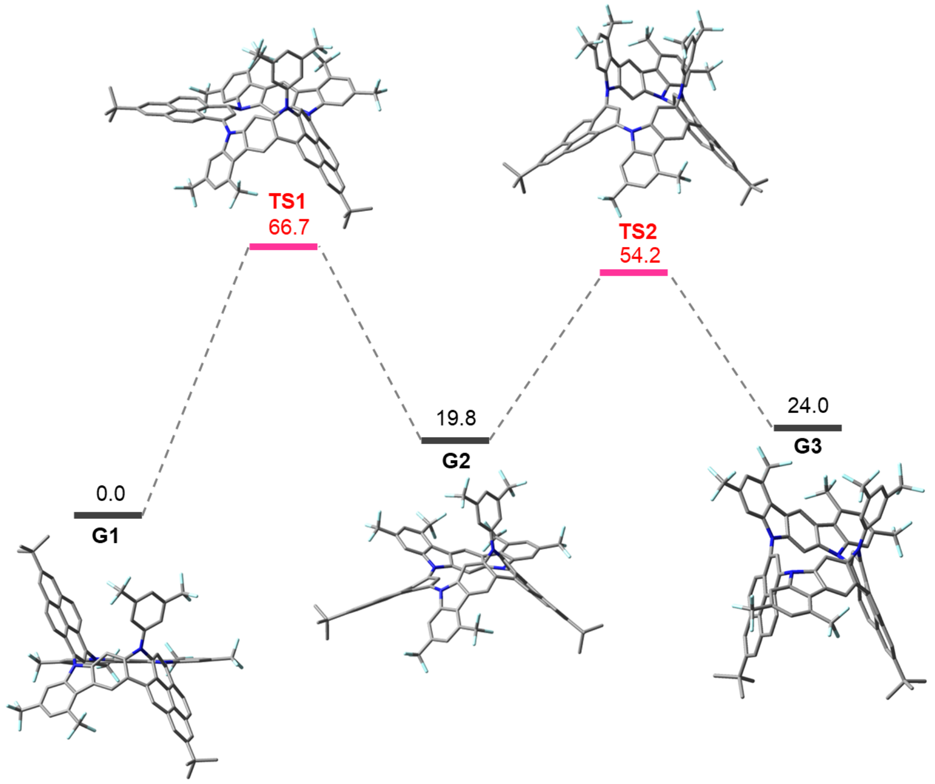

Figure S3. Calculated isomerization pathway for **MC3** with relative Gibbs free energy (kcal mol^{-1}) calculated at the B3LYP/6-31G(d) level.

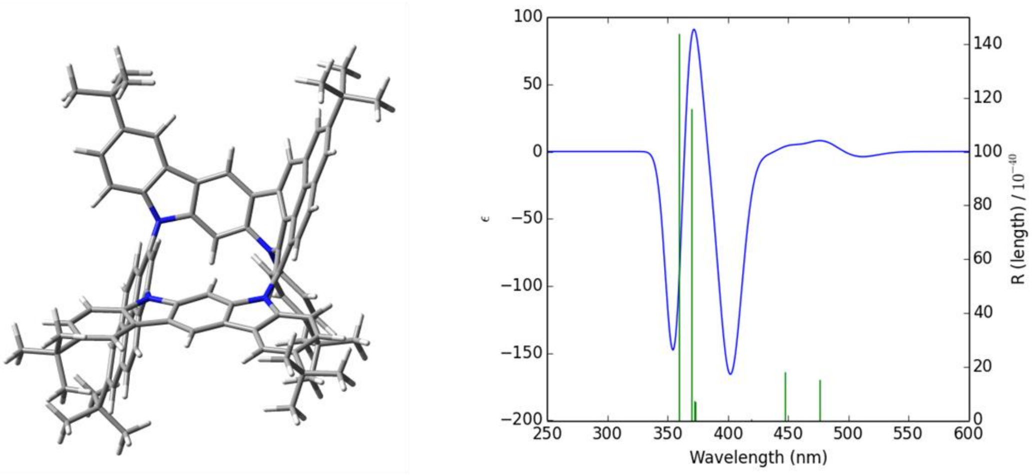

Figure S4. Optimized structure of **(+)MC1** (left) and its corresponding CD spectrum (right) calculated at the B3LYP/6-31G(d) level.

6. ^{1}H and ^{13}C NMR spectra

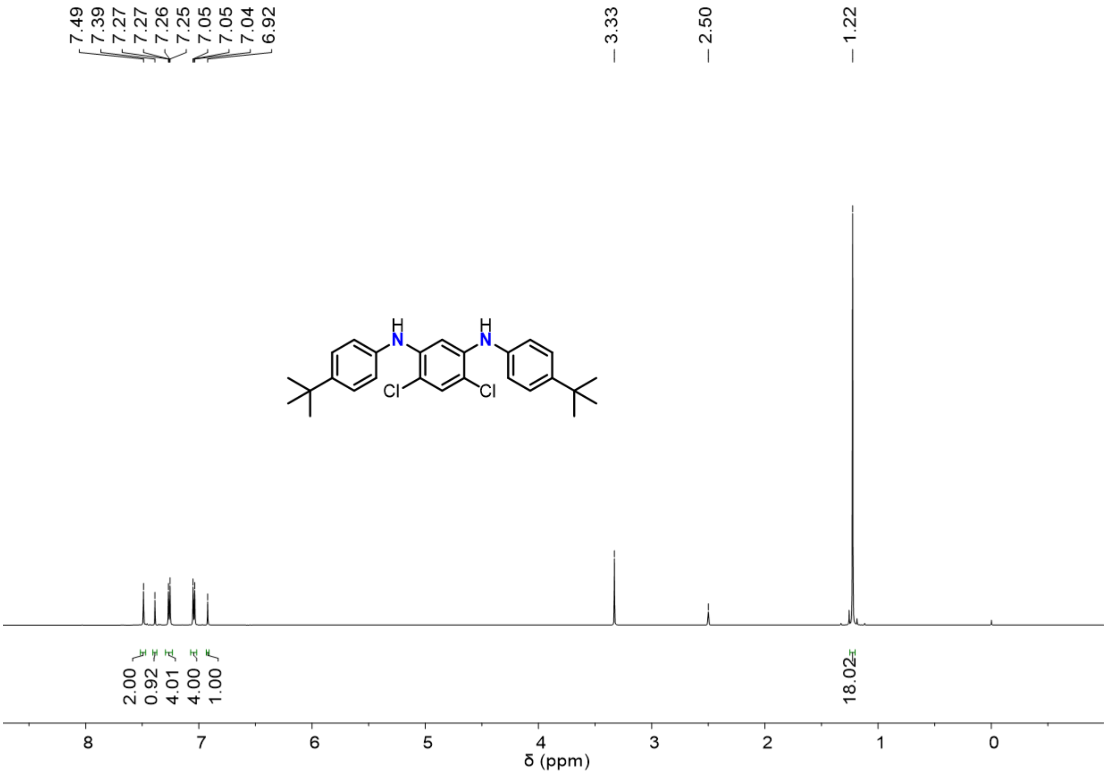

Figure S5. ^{1}H NMR spectrum (600 MHz) of compound **1a** in DMSO-d_6 at 298 K.

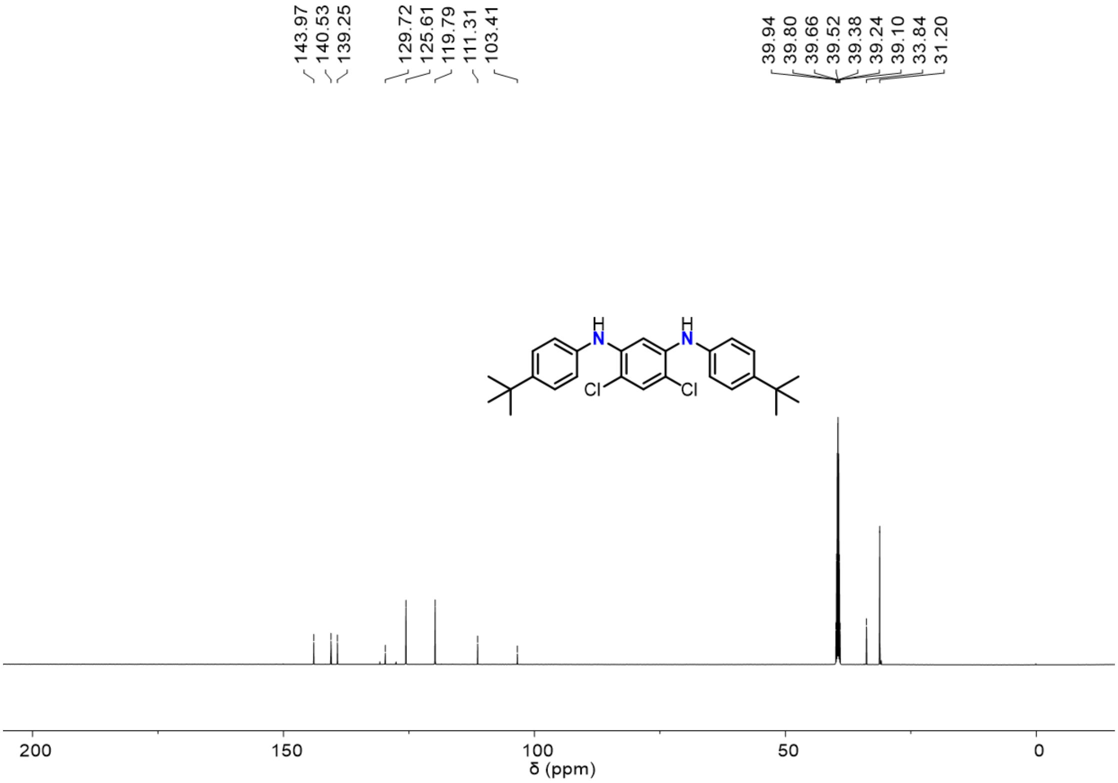

Figure S6. ^{13}C NMR spectrum (150 MHz) of compound **1a** in DMSO-d_6 at 298 K.

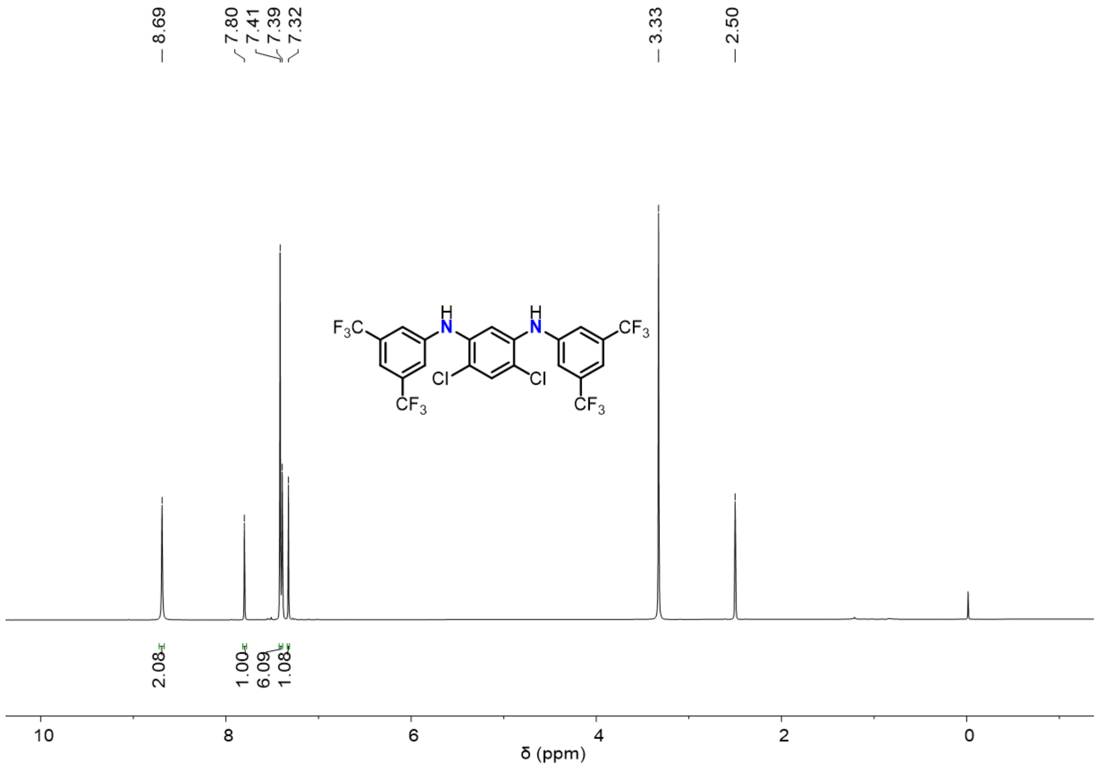

Figure S7. ^{1}H NMR spectrum (600 MHz) of **1b** in DMSO-d_6 at 298 K.

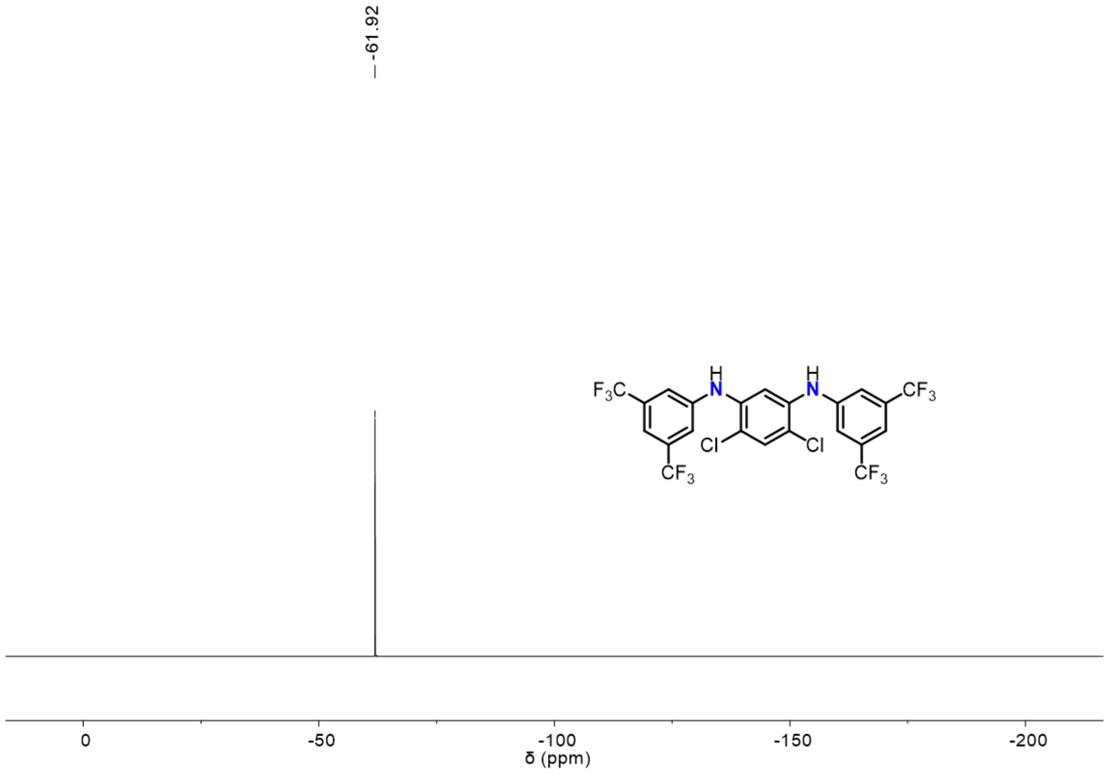

Figure S8. ^{19}F NMR spectrum (565 MHz) of compound **1b** in DMSO-d_6 at 298 K.

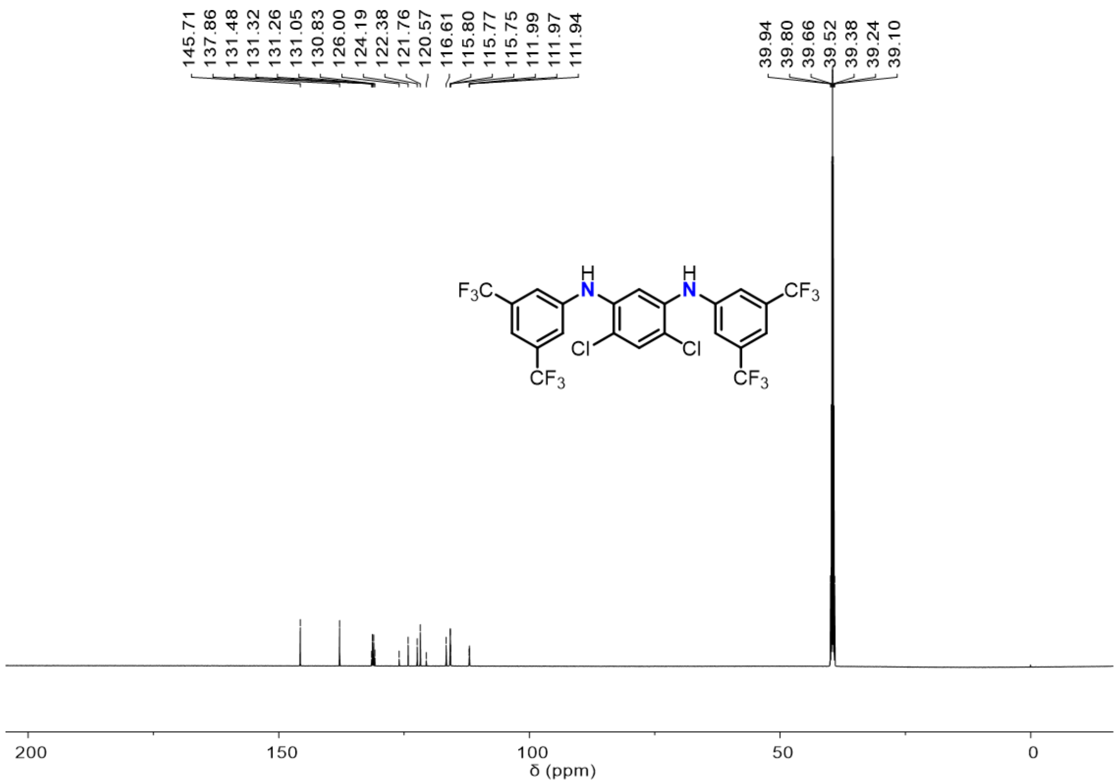

Figure S9. ^{13}C NMR spectrum (150 MHz) of **1b** in DMSO-d_6 at 298 K.

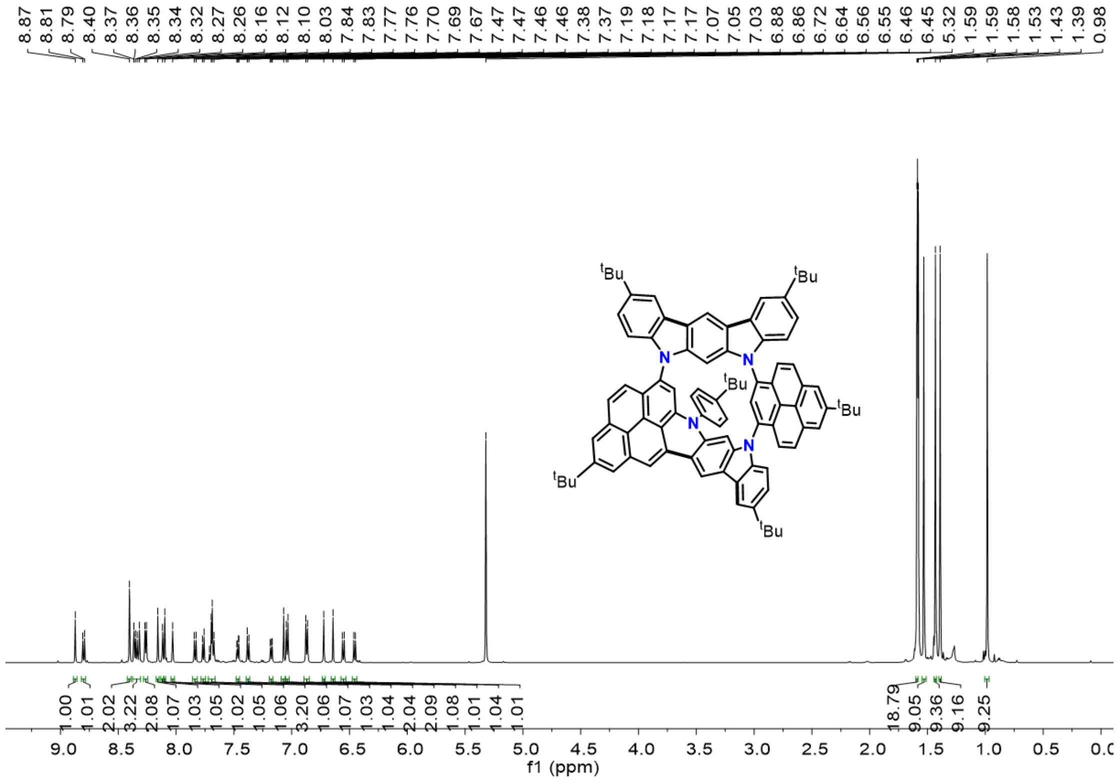

Figure S10. ^{1}H NMR spectrum (600 MHz) of compound **MC1** in CD_2Cl_2 at 298 K.

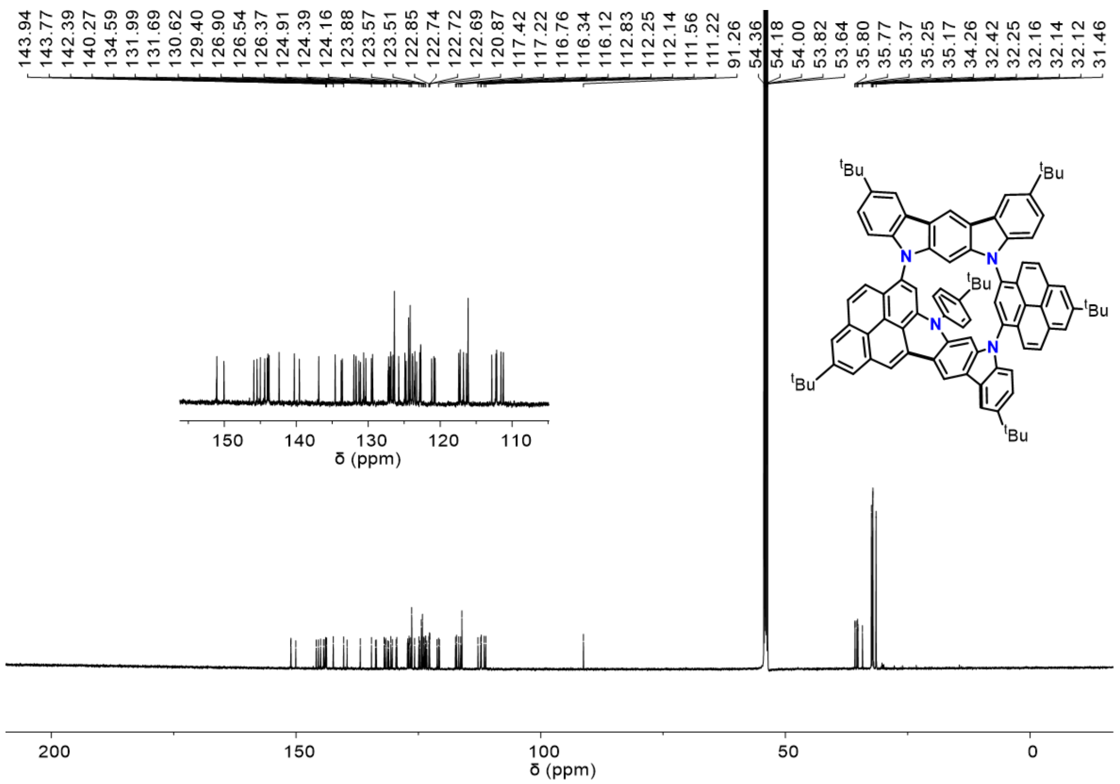

Figure S11. ^{13}C NMR spectrum (150 MHz) of compound **MC1** in CD_2Cl_2 at 298 K.

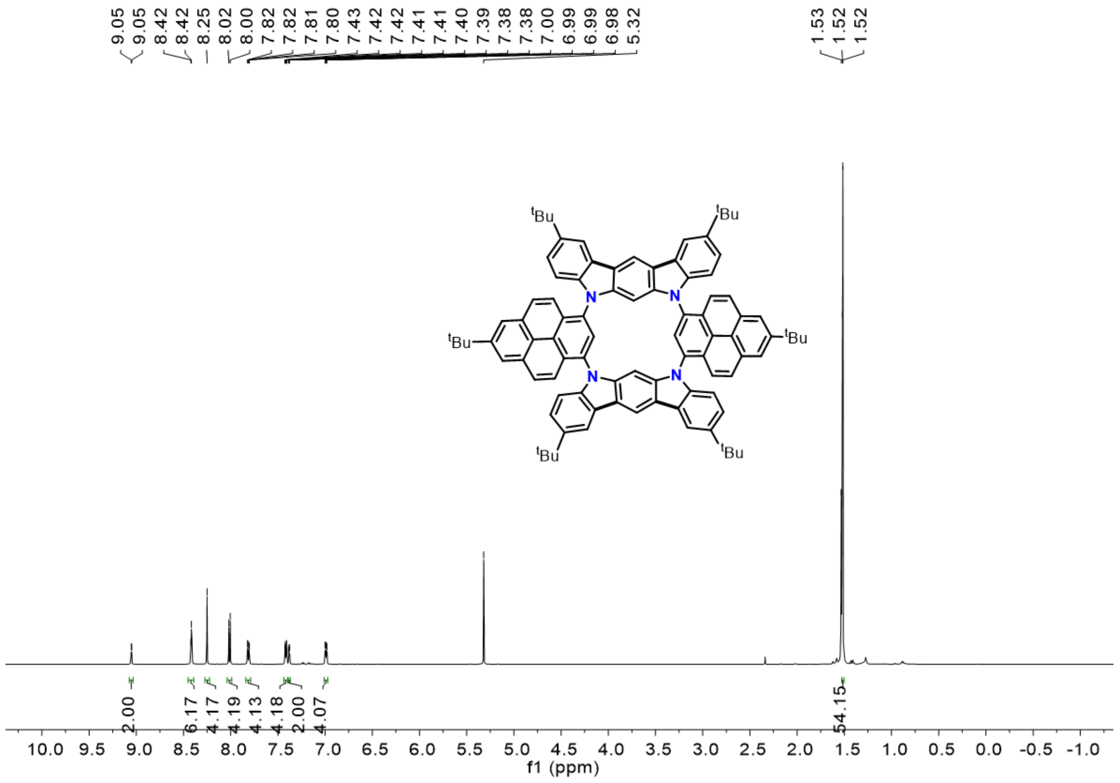

Figure S12. ^{1}H NMR spectrum (600 MHz) of compound **MC2** in CD_2Cl_2 at 298 K.

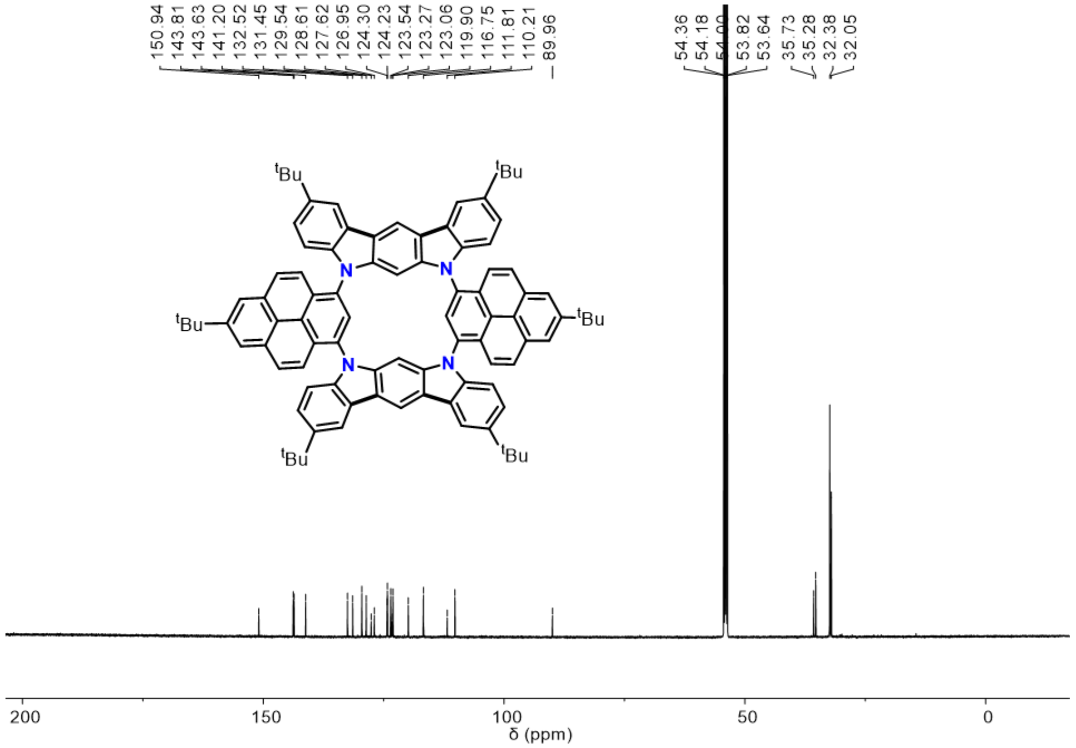

Figure S13. ^{13}C NMR spectrum (150 MHz) of compound **MC2** in CD_2Cl_2 at 298 K.

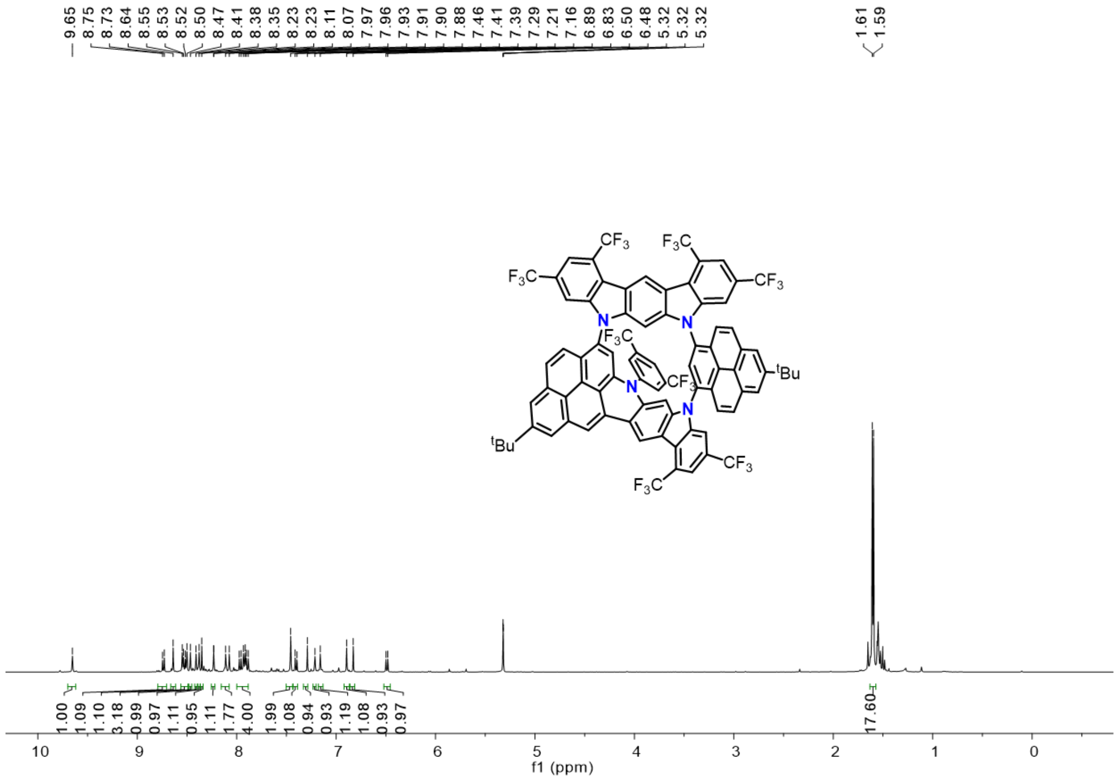

Figure S14. ^{1}H NMR spectrum (500 MHz) of **MC3** measured in CD_2Cl_2 at 298 K.

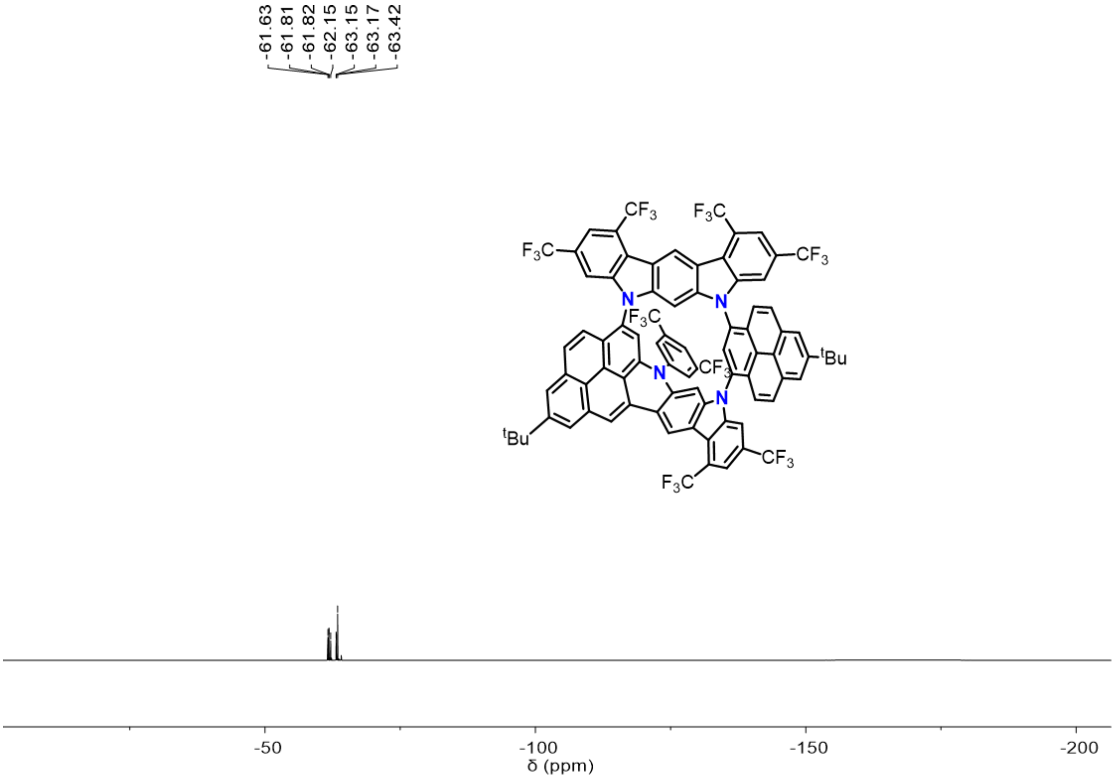

Figure S15. ^{19}F NMR spectrum (471 MHz) of **MC3** in CD_2Cl_2 at 298 K.

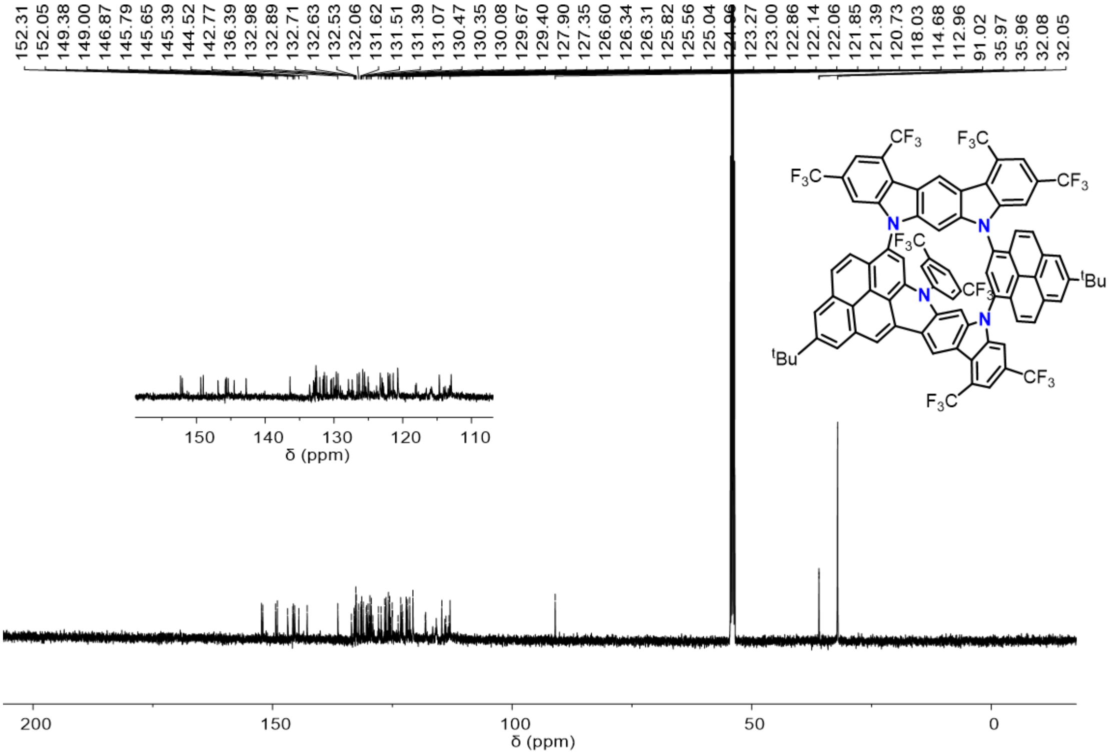

Figure S16. ^{13}C NMR spectrum (125 MHz) of **MC3** in CD_2Cl_2 at 298 K.

7. Mass spectra

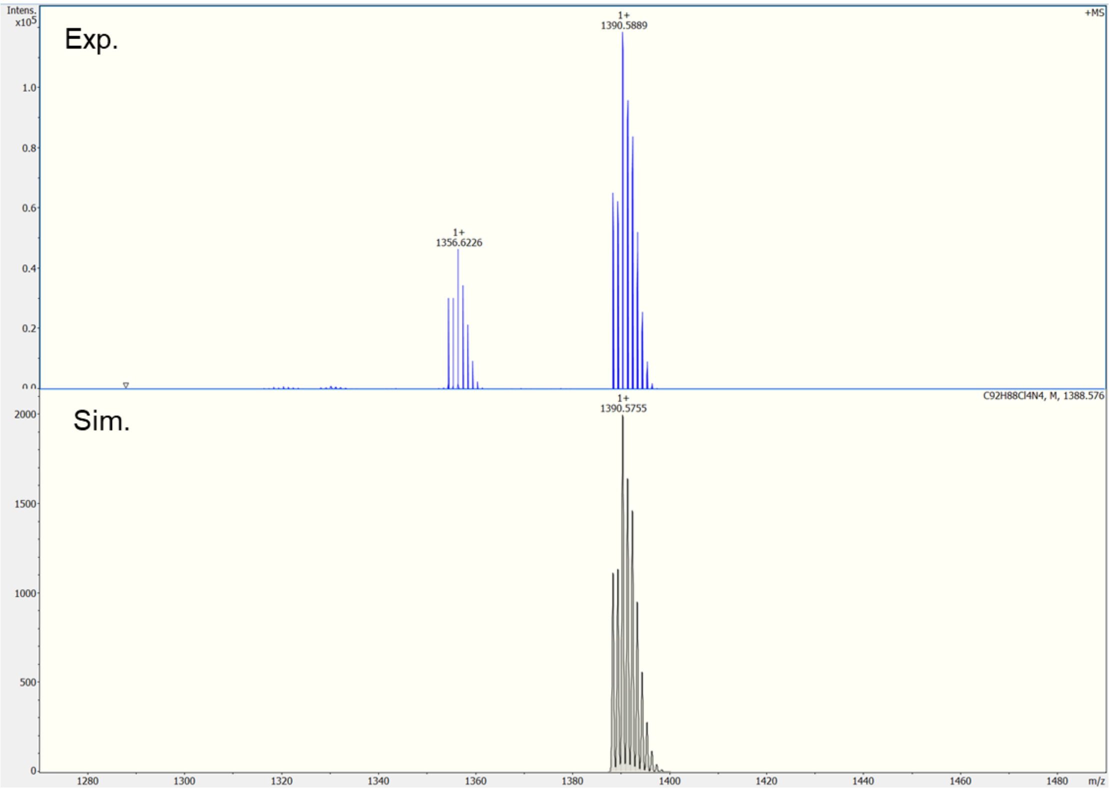

Figure S17. Mass spectrum (MALDI-TOF) of **3a**.

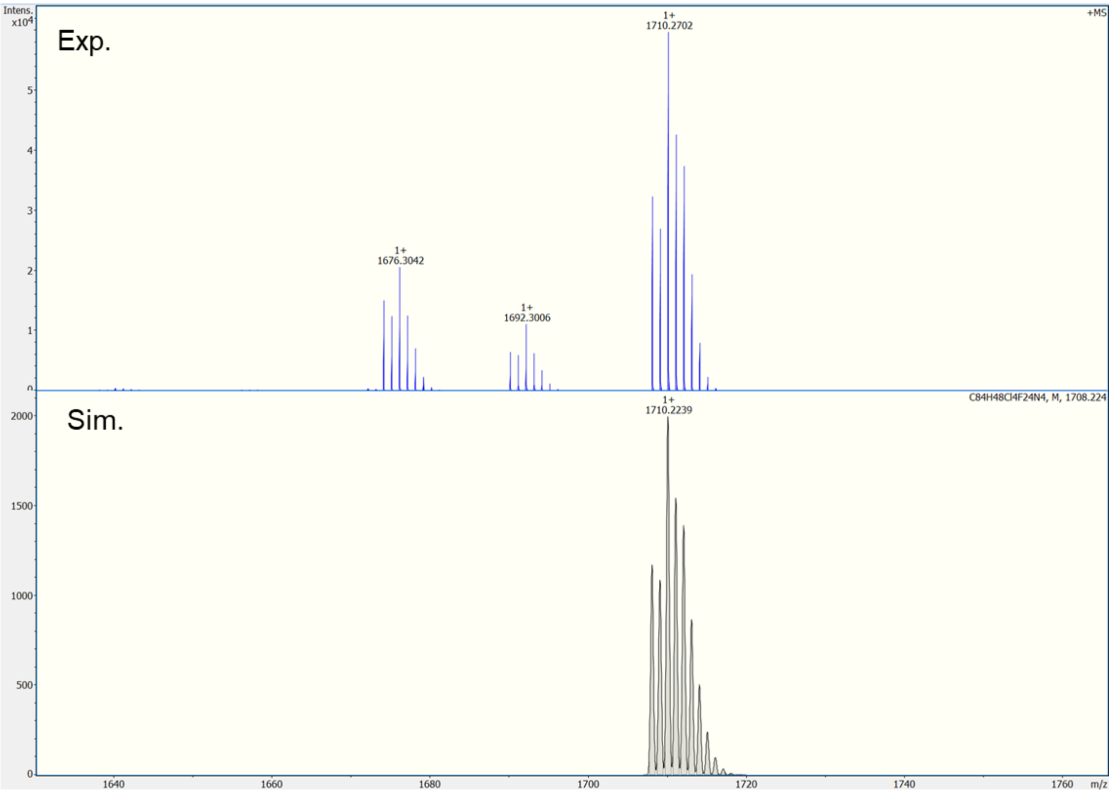

Figure S18. Mass spectrum (MALDI-TOF) of **3b**.

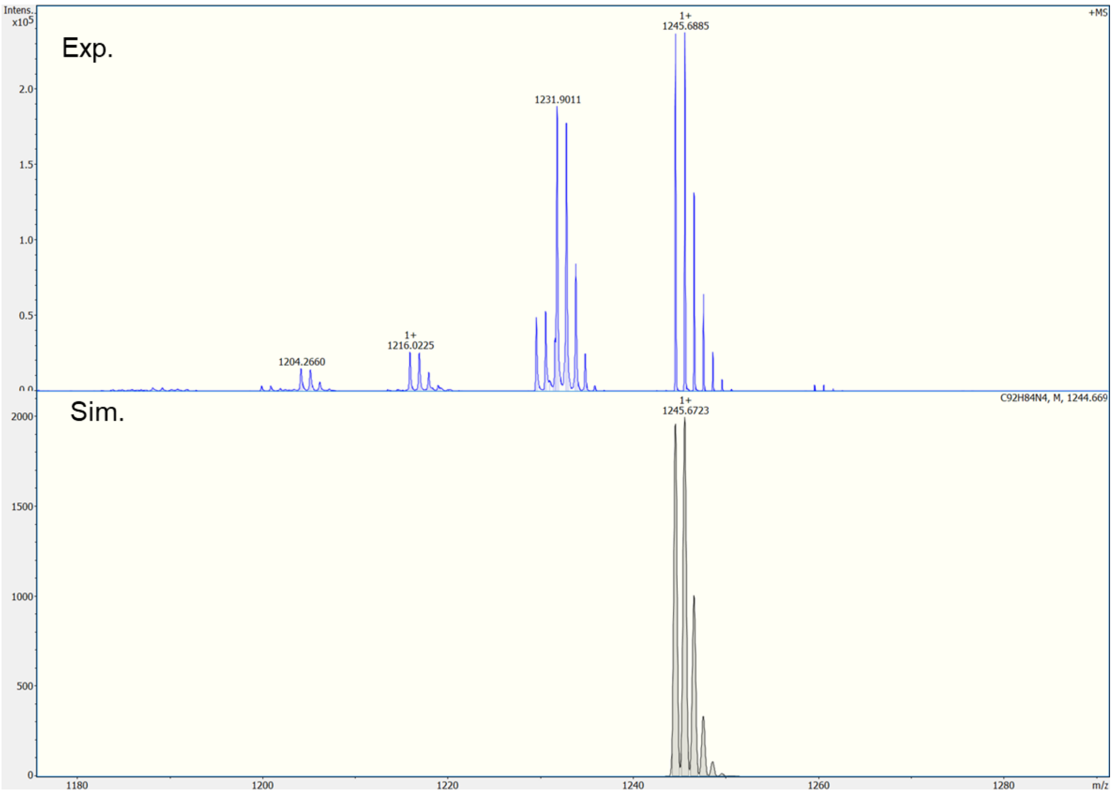

Figure S19. Mass spectrum (MALDI-TOF) of **MC1**.

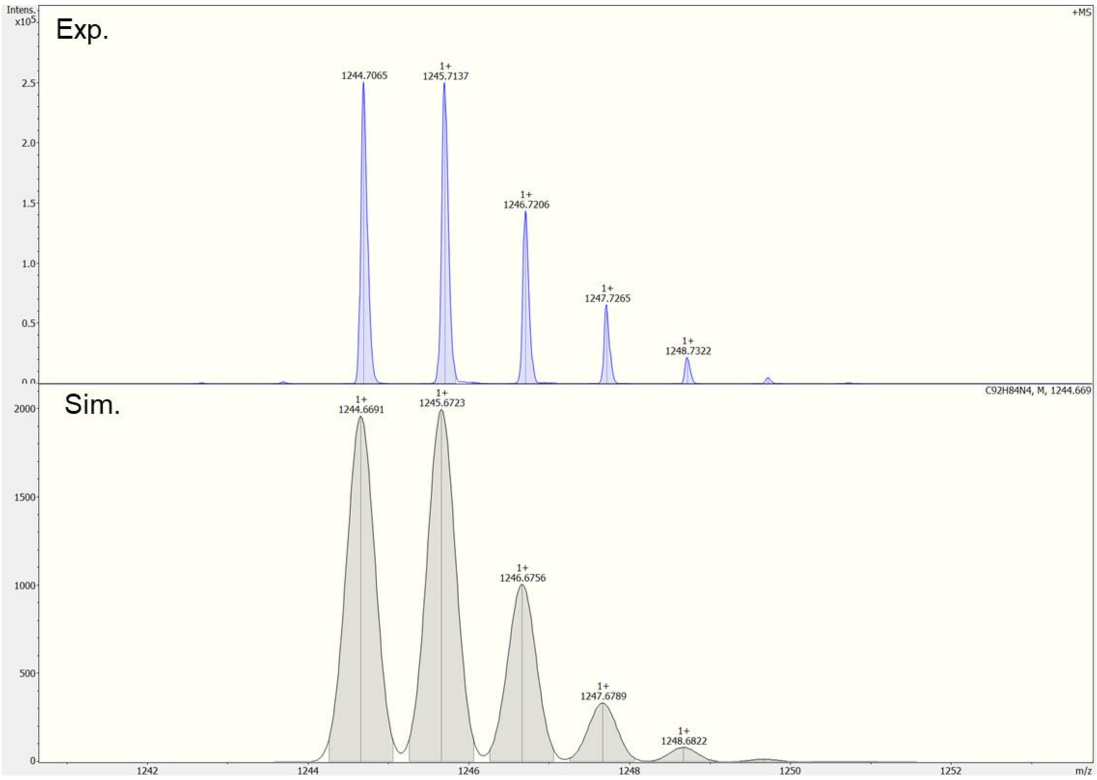

Figure S20. Mass spectrum (MALDI-TOF) of **MC2**.

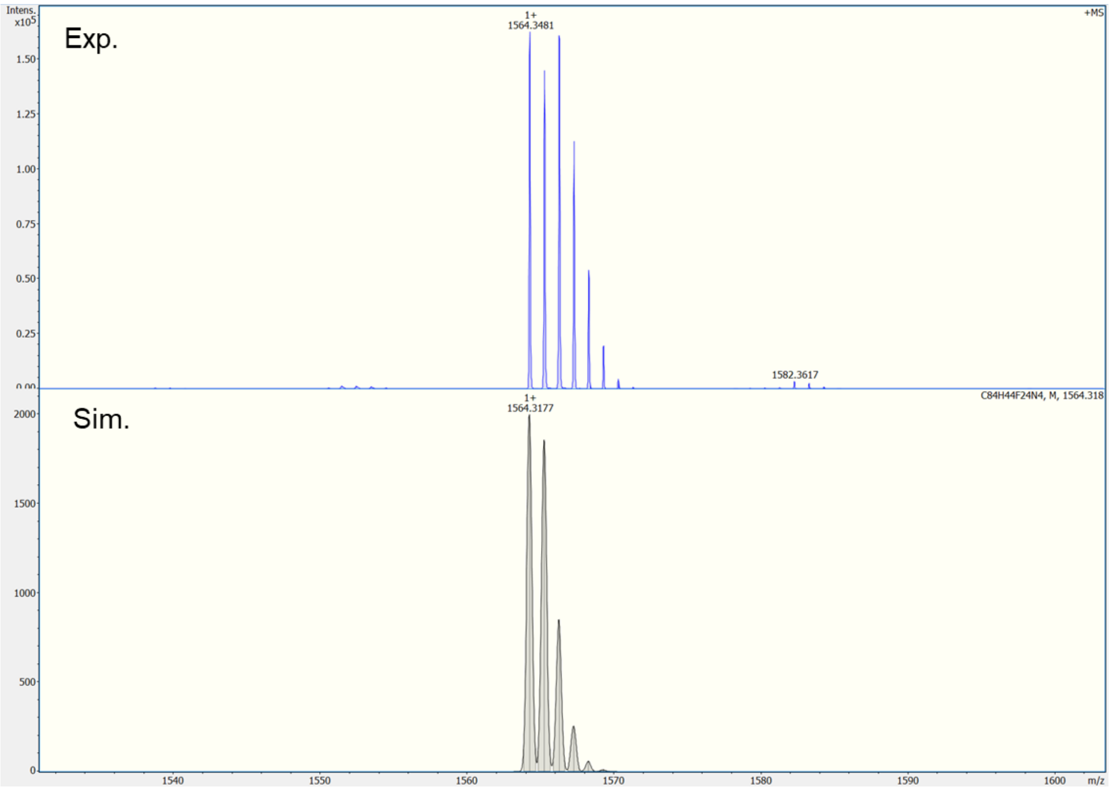

Figure S21. Mass spectrum (MALDI-TOF) of **MC3**.

8. References

[S1] Gaussian 16, Revision C.01, M. J. Frisch, G. W. Trucks, H. B. Schlegel, G. E. Scuseria, M. A. Robb, J. R. Cheeseman, G. Scalmani, V. Barone, G. A. Petersson, H. Nakatsuji, X. Li, M. Caricato, A. V. Marenich, J. Bloino, B. G. Janesko, R. Gomperts, B. Mennucci, H. P. Hratchian, J. V. Ortiz, A. F. Izmaylov, J. L. Sonnenberg, D. Williams-Young, F. Ding, F. Lipparini, F. Egidi, J. Goings, B. Peng, A. Petrone, T. Henderson, D. Ranasinghe, V. G. Zakrzewski, J. Gao, N. Rega, G. Zheng, W. Liang, M. Hada, M. Ehara, K. Toyota, R. Fukuda, J. Hasegawa, M. Ishida, T. Nakajima, Y. Honda, O. Kitao, H. Nakai, T. Vreven, K. Throssell, J. A. Montgomery, Jr., J. E. Peralta, F. Ogliaro, M. J. Bearpark, J. J. Heyd, E. N. Brothers, K. N. Kudin, V. N. Staroverov, T. A. Keith, R. Kobayashi, J. Normand, K. Raghavachari, A. P. Rendell, J. C. Burant, S. S. Iyengar, J. Tomasi, M. Cossi, J. M. Millam, M. Klene, C. Adamo, R. Cammi, J. W. Ochterski, R. L. Martin, K. Morokuma, O. Farkas, J. B. Foresman, and D. J. Fox, Gaussian, Inc., Wallingford CT, 2016.

[S2] Lu, T.; Chen, F. Multiwfn: A multifunctional wavefunction analyzer, *J. Comput. Chem.* **2012**, *33*, 580-592.

[S3] Humphrey, W.; Dalke, A.; Schulten, K., VMD - Visual Molecular Dynamics, *J. Molec. Graphics*, **1996**, *14*, 33-38
