# Supplementary material for: Synthesis of N-doped chiral macrocycles by regioselective palladium-catalyzed arylation
Source: Beilstein J Org Chem. 2025 Sep 15;21:1917–23. doi: 10.3762/bjoc.21.149 (PMC12456077; doi:10.3762/bjoc.21.149)

checkCIF/PLATON report

Structure factors have been supplied for datablock(s) cu_hku_mcp_tph_0m

THIS REPORT IS FOR GUIDANCE ONLY. IF USED AS PART OF A REVIEW PROCEDURE FOR PUBLICATION, IT SHOULD NOT REPLACE THE EXPERTISE OF AN EXPERIENCED CRYSTALLOGRAPHIC REFEREE.

No syntax errors found. CIF dictionary Interpreting this report

Datablock: cu_hku_mcp_tph_0m

```
Bond precision:   C-C = 0.0055 A           Wavelength=1.54178

Cell:            a=19.8177(5)      b=20.7758(5)      c=26.2065(6)
                 alpha=94.329(2)   beta=102.462(2)   gamma=116.553(1)
Temperature:     223 K
```

	Calculated	Reported
Volume	9240.0(4)	9240.0(4)
Space group	P -1	P -1
Hall group	-P 1	-P 1
Moiety formula	C92 H88 Cl4 N4 [+ solvent]	C92 H88 Cl4 N4
Sum formula	C92 H88 Cl4 N4 [+ solvent]	C92 H88 Cl4 N4
Mr	1391.47	1391.46
Dx,g cm-3	1.000	1.000
Z	4	4
Mu (mm-1)	1.471	1.471
F000	2944.0	2944.0
F000'	2956.63	
h,k,lmax	23,25,31	23,25,31
Nref	34056	33887
Tmin,Tmax	0.826,0.851	0.656,0.753
Tmin'	0.826	

```
Correction method= # Reported T Limits: Tmin=0.656 Tmax=0.753
AbsCorr = MULTI-SCAN

Data completeness= 0.995            Theta(max)= 68.560

R(reflections)= 0.0664( 22236)      wR2(reflections)= 0.2174( 33887)

S = 1.026                   Npar= 1928
```

The following ALERTS were generated. Each ALERT has the format
test-name_ALERT_alert-type_alert-level.
Click on the hyperlinks for more details of the test.

Alert level C

```
PLAT213_ALERT_2_C Atom C1B             has ADP max/min Ratio .....        3.2 oblate
PLAT220_ALERT_2_C NonSolvent   Resd 1  C   Ueq(max)/Ueq(min) Range        4.3 Ratio
PLAT220_ALERT_2_C NonSolvent   Resd 2  C   Ueq(max)/Ueq(min) Range        4.2 Ratio
PLAT222_ALERT_3_C NonSolvent Resd 1  H   Uiso(max)/Uiso(min) Range        5.4 Ratio
PLAT222_ALERT_3_C NonSolvent Resd 2  H   Uiso(max)/Uiso(min) Range        5.0 Ratio
PLAT230_ALERT_2_C Hirshfeld Test Diff for    C37      --C40      .        5.2 s.u.
PLAT234_ALERT_4_C Large Hirshfeld Difference C27      --C28A     .       0.20 Ang.
PLAT234_ALERT_4_C Large Hirshfeld Difference C27      --C30A     .       0.18 Ang.
PLAT234_ALERT_4_C Large Hirshfeld Difference C69      --C71      .       0.16 Ang.
PLAT234_ALERT_4_C Large Hirshfeld Difference C69      --C72      .       0.16 Ang.
PLAT234_ALERT_4_C Large Hirshfeld Difference C69      --C70A     .       0.17 Ang.
PLAT234_ALERT_4_C Large Hirshfeld Difference C69      --C71A     .       0.16 Ang.
PLAT234_ALERT_4_C Large Hirshfeld Difference C69      --C72A     .       0.18 Ang.
PLAT234_ALERT_4_C Large Hirshfeld Difference C170     --C1A      .       0.23 Ang.
PLAT234_ALERT_4_C Large Hirshfeld Difference C170     --C1B      .       0.19 Ang.
PLAT242_ALERT_2_C Low    'MainMol' Ueq as Compared to Neighbors of        C17 Check
PLAT242_ALERT_2_C Low    'MainMol' Ueq as Compared to Neighbors of        C27 Check
PLAT242_ALERT_2_C Low    'MainMol' Ueq as Compared to Neighbors of        C37 Check
PLAT242_ALERT_2_C Low    'MainMol' Ueq as Compared to Neighbors of        C53 Check
PLAT242_ALERT_2_C Low    'MainMol' Ueq as Compared to Neighbors of        C69 Check
PLAT242_ALERT_2_C Low    'MainMol' Ueq as Compared to Neighbors of        C89 Check
PLAT242_ALERT_2_C Low    'MainMol' Ueq as Compared to Neighbors of       C113 Check
PLAT242_ALERT_2_C Low    'MainMol' Ueq as Compared to Neighbors of       C123 Check
PLAT242_ALERT_2_C Low    'MainMol' Ueq as Compared to Neighbors of       C139 Check
PLAT242_ALERT_2_C Low    'MainMol' Ueq as Compared to Neighbors of       C170 Check
PLAT242_ALERT_2_C Low    'MainMol' Ueq as Compared to Neighbors of       C182 Check
PLAT340_ALERT_3_C Low Bond Precision on  C-C Bonds ...............    0.00545 Ang.
PLAT906_ALERT_3_C Large K Value in the Analysis of Variance ......      3.590 Check
PLAT911_ALERT_3_C Missing FCF Refl Between Thmin & STh/L=    0.600         63 Report
                 4 -4  1,  -1  0  1,  -9 24  1,   1 -2  2,  -1  1  2, -10 10  2,
                 1 -3  3,   0 -1  3,   1 -1  3,   1  0  3,  -7  6  3,  -4  7  3,
                -8 24  3,   1  0  4,   4  4  4,   2 -1  5,   2  1  5,   2  2  5,
                -2-11  6,   0 -4  6,   2 -1  6,   3 -1  6,   2  0  6,   2  2  6,
                 3 -2  7,  -4 -1  7,  -3 -1  7,   2 -1  7,   4  0  7,  -1 -4  8,
                -2 -2  8,   1 -1  8,   3 -2  9,   4 -6 10,  -1  0 10,  11 -4 12,
                 6-20 13,  17-19 13,  11 -4 13,  16-19 14,  10-16 14,  12-14 15,
                -8 17 15,   9-20 16,  -8 17 16,   4 10 17,  13-19 18,   9 -7 19,
                 2 -9 20,   7 -5 20,  -2 -3 23,  10 -2 23,  10 -1 23,   3  2 23,
                 9 -1 24,   8 -1 25, -15  2 25, -14  5 26, -13 -3 28, -12 -3 28,
               -11 -3 28, -12 -1 28, -14  1 28,
PLAT918_ALERT_3_C Reflection(s) with I(obs) much Smaller I(calc) .          1 Check
```

Alert level G

```
PLAT002_ALERT_2_G Number of Distance or Angle Restraints on AtSite         21 Note
PLAT003_ALERT_2_G Number of Uiso or U(i,j) Restrained non-H Atoms          18 Report
PLAT072_ALERT_2_G SHELXL First  Parameter in WGHT  Unusually Large       0.12 Report
PLAT176_ALERT_4_G The CIF-Embedded .res File Contains SADI Records          9 Report
PLAT178_ALERT_4_G The CIF-Embedded .res File Contains SIMU Records          3 Report
PLAT187_ALERT_4_G The CIF-Embedded .res File Contains RIGU Records          6 Report
```

```
PLAT188_ALERT_3_G A Non-default SIMU Restraint Value has been used     0.0100 Report
PLAT188_ALERT_3_G A Non-default SIMU Restraint Value has been used     0.0100 Report
PLAT188_ALERT_3_G A Non-default SIMU Restraint Value has been used     0.0100 Report
PLAT190_ALERT_3_G A Non-default RIGU Restraint Value for First Par     0.0100 Report
PLAT190_ALERT_3_G A Non-default RIGU Restraint Value for SecondPar     0.0200 Report
PLAT190_ALERT_3_G A Non-default RIGU Restraint Value for First Par     0.0100 Report
PLAT190_ALERT_3_G A Non-default RIGU Restraint Value for SecondPar     0.0200 Report
PLAT190_ALERT_3_G A Non-default RIGU Restraint Value for First Par     0.0100 Report
PLAT190_ALERT_3_G A Non-default RIGU Restraint Value for SecondPar     0.0200 Report
PLAT190_ALERT_3_G A Non-default RIGU Restraint Value for First Par     0.0100 Report
PLAT190_ALERT_3_G A Non-default RIGU Restraint Value for SecondPar     0.0200 Report
PLAT190_ALERT_3_G A Non-default RIGU Restraint Value for First Par     0.0100 Report
PLAT190_ALERT_3_G A Non-default RIGU Restraint Value for SecondPar     0.0200 Report
PLAT190_ALERT_3_G A Non-default RIGU Restraint Value for First Par     0.0100 Report
PLAT190_ALERT_3_G A Non-default RIGU Restraint Value for SecondPar     0.0200 Report
PLAT301_ALERT_3_G Main Residue  Disorder .............(Resd    1)         6% Note
PLAT301_ALERT_3_G Main Residue  Disorder .............(Resd    2)         3% Note
PLAT333_ALERT_2_G Large Aver C6-Ring C-C Dist C75      -C84      .       1.42 Ang.
PLAT412_ALERT_2_G Short Intra XH3 .. XHn     H23      ..H29F     .       1.86 Ang.
                                                    x,y,z  =     1_555 Check
PLAT412_ALERT_2_G Short Intra XH3 .. XHn     H25      ..H30D     .       2.14 Ang.
                                                    x,y,z  =     1_555 Check
PLAT412_ALERT_2_G Short Intra XH3 .. XHn     H16L     ..H164     .       2.05 Ang.
                                                    x,y,z  =     1_555 Check
PLAT412_ALERT_2_G Short Intra XH3 .. XHn     H164     ..H18J     .       1.71 Ang.
                                                    x,y,z  =     1_555 Check
PLAT434_ALERT_2_G Short Inter HL..HL Contact Cl3      ..Cl8      .       3.38 Ang.
                                                    x,y,z  =     1_555 Check
PLAT606_ALERT_4_G Solvent Accessible VOID(S) in Structure .......          ! Info
PLAT720_ALERT_4_G Number of Unusual/Non-Standard Labels .........          6 Note
              H1AA    H1AB    H1AC    H1BA    H1BB    H1BC
PLAT721_ALERT_1_G Bond    Calc     0.96000, Rep     0.97000 Dev...       0.01 Ang.
              C71A     -H71D           1_555   1_555 ........     #  414 Check
PLAT721_ALERT_1_G Bond    Calc     0.96000, Rep     0.97000 Dev...       0.01 Ang.
              C70A     -H70D           1_555   1_555 ........     #  420 Check
PLAT721_ALERT_1_G Bond    Calc     0.96000, Rep     0.97000 Dev...       0.01 Ang.
              C30A     -H30D           1_555   1_555 ........     #  426 Check
PLAT860_ALERT_3_G Number of Least-Squares Restraints ............        219 Note
PLAT868_ALERT_4_G ALERTS Due to the Use of _smtbx_masks Suppressed         ! Info
PLAT910_ALERT_3_G Missing # of FCF Reflection(s) Below Theta(Min).         1 Note
                0  0  1,
PLAT912_ALERT_4_G Missing # of FCF Reflections Above STh/L=  0.600       105 Note
PLAT913_ALERT_3_G Missing # of Very Strong Reflections in FCF ....         1 Note
               -1  0  1,
PLAT933_ALERT_2_G Number of HKL-OMIT Records in Embedded .res File        16 Note
               -1 -4  8,   1 -3  3,  -7  6  3,  -2 -2  8,  -4 -1  7,   0 -1  3,
                2  2  5,   1 -1  3,  -1  0 10,   4  4  4,   4 -6 10,   1 -2  2,
               -3 -1  7,   1 -1  8,   0 -4  6,  -4  7  3,
PLAT941_ALERT_3_G Average HKL Measurement Multiplicity ..........        3.5 Low
PLAT969_ALERT_5_G The 'Henn et al.' R-Factor-gap value ..........      3.870 Note
              Predicted wR2: Based on SigI**2  5.62 or SHELX Weight 21.18
PLAT978_ALERT_2_G Number C-C Bonds with Positive Residual Density.         1 Info
```

---

0 **ALERT level A** = Most likely a serious problem - resolve or explain

0 **ALERT level B** = A potentially serious problem, consider carefully

30 **ALERT level C** = Check. Ensure it is not caused by an omission or oversight

```
43 ALERT level G = General information/check it is not something unexpected

 3 ALERT type 1 CIF construction/syntax error, inconsistent or missing data
26 ALERT type 2 Indicator that the structure model may be wrong or deficient
27 ALERT type 3 Indicator that the structure quality may be low
16 ALERT type 4 Improvement, methodology, query or suggestion
 1 ALERT type 5 Informative message, check
```

It is advisable to attempt to resolve as many as possible of the alerts in all categories. Often the minor alerts point to easily fixed oversights, errors and omissions in your CIF or refinement strategy, so attention to these fine details can be worthwhile. In order to resolve some of the more serious problems it may be necessary to carry out additional measurements or structure refinements. However, the purpose of your study may justify the reported deviations and the more serious of these should normally be commented upon in the discussion or experimental section of a paper or in the "special_details" fields of the CIF. checkCIF was carefully designed to identify outliers and unusual parameters, but every test has its limitations and alerts that are not important in a particular case may appear. Conversely, the absence of alerts does not guarantee there are no aspects of the results needing attention. It is up to the individual to critically assess their own results and, if necessary, seek expert advice.

Publication of your CIF in IUCr journals

A basic structural check has been run on your CIF. These basic checks will be run on all CIFs submitted for publication in IUCr journals (*Acta Crystallographica*, *Journal of Applied Crystallography*, *Journal of Synchrotron Radiation*); however, if you intend to submit to *Acta Crystallographica Section C* or *E* or *IUCrData*, you should make sure that full publication checks are run on the final version of your CIF prior to submission.

Publication of your CIF in other journals

Please refer to the *Notes for Authors* of the relevant journal for any special instructions relating to CIF submission.

PLATON version of 22/08/2024; check.def file version of 21/08/2024

Datablock cu_hku_mcp_tph_0m - ellipsoid plot

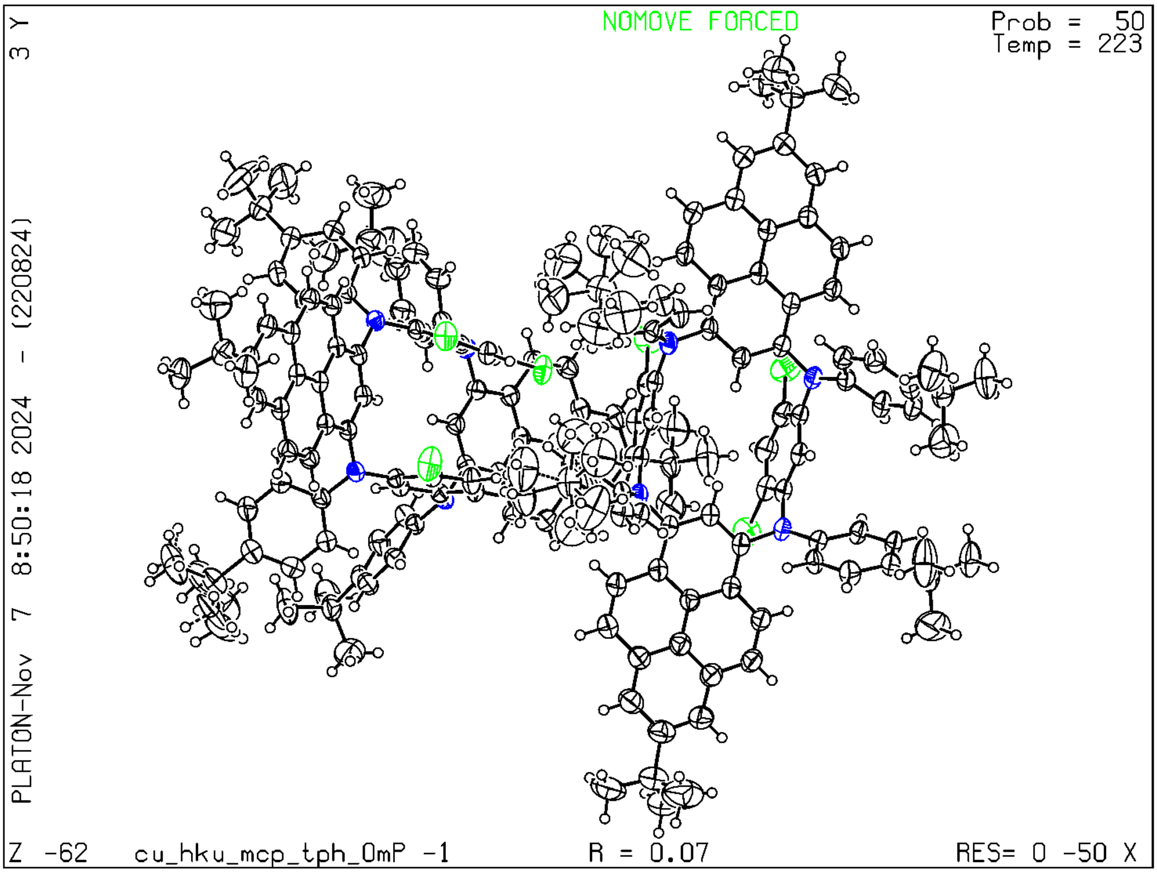

Supplement: File 2 — Crystallographic information files for compounds 3a, MC2, and MC3. [file Beilstein_J_Org_Chem-21-1917-s002.zip › 3a_cifreport.pdf]
