# Supplementary material for: Synthesis of N-doped chiral macrocycles by regioselective palladium-catalyzed arylation
Source: Beilstein J Org Chem. 2025 Sep 15;21:1917–23. doi: 10.3762/bjoc.21.149 (PMC12456077; doi:10.3762/bjoc.21.149)

checkCIF/PLATON report

Structure factors have been supplied for datablock(s) cu_hku_dicz_p2_0m

THIS REPORT IS FOR GUIDANCE ONLY. IF USED AS PART OF A REVIEW PROCEDURE FOR PUBLICATION, IT SHOULD NOT REPLACE THE EXPERTISE OF AN EXPERIENCED CRYSTALLOGRAPHIC REFEREE.

No syntax errors found. CIF dictionary Interpreting this report

Datablock: cu_hku_dicz_p2_0m

Bond precision: C-C = 0.0074 A Wavelength=1.54178

Cell:	a=14.6091(3)	b=15.3776(4)	c=22.3624(5)
	alpha=92.706(2)	beta=102.868(2)	gamma=107.854(2)
Temperature:	223 K		

	Calculated	Reported
Volume	4625.5(2)	4625.50(19)
Space group	P -1	P -1
Hall group	-P 1	-P 1
Moiety formula	C92 H84 N4 [+ solvent]	C92 H84 N4
Sum formula	C92 H84 N4 [+ solvent]	C92 H84 N4
Mr	1245.64	1245.63
Dx,g cm-3	0.894	0.894
Z	2	2
Mu (mm-1)	0.390	0.390
F000	1328.0	1328.0
F000’	1331.38	
h,k,lmax	17,18,26	17,18,26
Nref	16352	16257
Tmin,Tmax	0.951,0.958	0.533,0.753
Tmin’	0.951	

Correction method= # Reported T Limits: Tmin=0.533 Tmax=0.753
AbsCorr = MULTI-SCAN

Data completeness= 0.994 Theta(max)= 66.593

R(reflections)= 0.1085(10661) wR2(reflections)= 0.2761(16257)

S = 1.001 Npar= 914

The following ALERTS were generated. Each ALERT has the format
test-name_ALERT_alert-type_alert-level.
Click on the hyperlinks for more details of the test.

Alert level C

```
DIFMX02_ALERT_1_C  The maximum difference density is > 0.1*ZMAX*0.75
            The relevant atom site should be identified.
PLAT082_ALERT_2_C High R1 Value ................................       0.11 Report
PLAT084_ALERT_3_C High wR2 Value (i.e. > 0.25) ..................       0.28 Report
PLAT097_ALERT_2_C Large Reported Max.  (Positive) Residual Density       0.70 eA-3
PLAT220_ALERT_2_C NonSolvent   Resd 1  C   Ueq(max)/Ueq(min) Range        3.3 Ratio
PLAT222_ALERT_3_C NonSolvent Resd 1  H   Uiso(max)/Uiso(min) Range        4.1 Ratio
PLAT234_ALERT_4_C Large Hirshfeld Difference C89      --C91      .       0.17 Ang.
PLAT234_ALERT_4_C Large Hirshfeld Difference C89      --C90A     .       0.19 Ang.
PLAT242_ALERT_2_C Low    'MainMol' Ueq as Compared to Neighbors of        C17 Check
PLAT242_ALERT_2_C Low    'MainMol' Ueq as Compared to Neighbors of        C39 Check
PLAT242_ALERT_2_C Low    'MainMol' Ueq as Compared to Neighbors of        C43 Check
PLAT242_ALERT_2_C Low    'MainMol' Ueq as Compared to Neighbors of        C85 Check
PLAT242_ALERT_2_C Low    'MainMol' Ueq as Compared to Neighbors of        C89 Check
PLAT340_ALERT_3_C Low Bond Precision on  C-C Bonds ..............    0.00743 Ang.
PLAT906_ALERT_3_C Large K Value in the Analysis of Variance ......      5.465 Check
PLAT906_ALERT_3_C Large K Value in the Analysis of Variance ......      2.481 Check
PLAT911_ALERT_3_C Missing FCF Refl Between Thmin & STh/L=    0.595         95 Report
               -3  1  0,  -2  1  0,  -1  1  0,   0  1  0,  -3  2  0,   0  3  0,
               -1  4  0, -12 11  0,   8-18  1,   5 -9  1,   2 -4  1,   1 -3  1,
                5 -3  1,   2 -2  1,   3 -2  1,   4 -2  1,   2 -1  1,  -2  2  1,
                2  2  1,  -4  3  1,  -2  3  1,  -2  4  1,   1  5  1,  -6  8  1,
                2  8  1,   8-18  2,  -2 -7  2,  -3 -6  2,   0 -3  2,   1 -3  2,
                4 -3  2,  -5 -2  2,   1 -2  2,   0  1  2,   2  3  2,  -3 14  2,
                4 -9  3,  -1  0  3,  -1  1  3,   2  1  3,   3  1  3,   3  2  3,
               -2  3  3,   1  5  3,   2  5  3,   1  6  3,   3 -7  4,   1 -2  4,
                1 -1  4,  -2  2  4,  10  9  4,   9 10  4,   6 12  4,   2 -7  5,
                1 -3  5,   1  0  5,  -1  1  5,   3  1  5,   3  2  5,  -9  4  5,
               -7  7  5,  10  9  5,   9 10  5,   3 -7  6,   6 -3  6,   2  2  6,
               -4  3  6,  -7  7  6,   3 -8  7,   7 11  7,   4 -7  8,  15 -6  8,
               -5  1  8,  -8  2  8,   8 10  8,   7 11  8,   6 12  8,  -8  5  9,
                6 11 10,   2 -5 11,  -1  0 11,  10 -7 12,   6 10 12,   5 11 12,
              -11 13 13, -16  4 15,   4 10 15, -15  5 17, -15  6 17, -15  3 18,
              -15  4 18, -15  5 18, -13  2 19, -11 -4 21,   2 -3 25,
PLAT918_ALERT_3_C Reflection(s) with I(obs) much Smaller I(calc) .          1 Check
```

Alert level G

```
PLAT002_ALERT_2_G Number of Distance or Angle Restraints on AtSite          7 Note
PLAT003_ALERT_2_G Number of Uiso or U(i,j) Restrained non-H Atoms           6 Report
PLAT083_ALERT_2_G SHELXL Second Parameter in WGHT  Unusually Large      20.00 Why ?
PLAT154_ALERT_1_G The s.u.'s on the Cell Angles are Equal ..(Note)      0.002 Degree
PLAT172_ALERT_4_G The CIF-Embedded .res File Contains DFIX Records          1 Report
PLAT176_ALERT_4_G The CIF-Embedded .res File Contains SADI Records          3 Report
PLAT178_ALERT_4_G The CIF-Embedded .res File Contains SIMU Records          1 Report
PLAT187_ALERT_4_G The CIF-Embedded .res File Contains RIGU Records          2 Report
PLAT188_ALERT_3_G A Non-default SIMU Restraint Value has been used     0.0100 Report
PLAT190_ALERT_3_G A Non-default RIGU Restraint Value for First Par     0.0100 Report
PLAT190_ALERT_3_G A Non-default RIGU Restraint Value for SecondPar     0.0200 Report
PLAT190_ALERT_3_G A Non-default RIGU Restraint Value for First Par     0.0100 Report
```

```
PLAT190_ALERT_3_G A Non-default RIGU Restraint Value for SecondPar     0.0200 Report
PLAT301_ALERT_3_G Main Residue  Disorder ..............(Resd    1)         3% Note
PLAT412_ALERT_2_G Short Intra XH3 .. XHn      H82       ..H92F     .       2.08 Ang.
                                                         x,y,z  =     1_555 Check
PLAT412_ALERT_2_G Short Intra XH3 .. XHn      H84       ..H90E     .       1.91 Ang.
                                                         x,y,z  =     1_555 Check
PLAT606_ALERT_4_G Solvent Accessible VOID(S) in Structure ........           ! Info
PLAT860_ALERT_3_G Number of Least-Squares Restraints ............          72 Note
PLAT868_ALERT_4_G ALERTS Due to the Use of _smtbx_masks Suppressed           ! Info
PLAT909_ALERT_3_G Percentage of I>2sig(I) Data at Theta(Max) Still         39% Note
PLAT913_ALERT_3_G Missing # of Very Strong Reflections in FCF ....           1 Note
                0  1  2,
PLAT933_ALERT_2_G Number of HKL-OMIT Records in Embedded .res File          29 Note
                1  5  3,   3  1  3,  -1  1  3,  -1  0  3,  -5 -2  2,   2  2  1,
                0  3  0,  -2  3  3,   3  2  5,  -4  3  6,  -1  0 11,  -5  1  8,
                3 -7  4,   2  8  1,   3  1  5,   6 -3  6,   2 -2  1,   2  3  2,
                3 -7  6,   1  5  1,  -3 -6  2,   3  2  3,   2  5  3,   1  6  3,
                2  2  6,  -2 -7  2,   1 -1  4,  -1  1  5,  -2  4  1,
PLAT941_ALERT_3_G Average HKL Measurement Multiplicity ..........         3.6 Low
PLAT969_ALERT_5_G The 'Henn et al.' R-Factor-gap value ..........       3.053 Note
              Predicted wR2: Based on SigI**2  9.04 or SHELX Weight 27.59
PLAT978_ALERT_2_G Number C-C Bonds with Positive Residual Density.           0 Info
```

```
   0 ALERT level A = Most likely a serious problem - resolve or explain
   0 ALERT level B = A potentially serious problem, consider carefully
  18 ALERT level C = Check. Ensure it is not caused by an omission or oversight
  25 ALERT level G = General information/check it is not something unexpected

   2 ALERT type 1 CIF construction/syntax error, inconsistent or missing data
  15 ALERT type 2 Indicator that the structure model may be wrong or deficient
  17 ALERT type 3 Indicator that the structure quality may be low
   8 ALERT type 4 Improvement, methodology, query or suggestion
   1 ALERT type 5 Informative message, check
```

It is advisable to attempt to resolve as many as possible of the alerts in all categories. Often the minor alerts point to easily fixed oversights, errors and omissions in your CIF or refinement strategy, so attention to these fine details can be worthwhile. In order to resolve some of the more serious problems it may be necessary to carry out additional measurements or structure refinements. However, the purpose of your study may justify the reported deviations and the more serious of these should normally be commented upon in the discussion or experimental section of a paper or in the "special_details" fields of the CIF. checkCIF was carefully designed to identify outliers and unusual parameters, but every test has its limitations and alerts that are not important in a particular case may appear. Conversely, the absence of alerts does not guarantee there are no aspects of the results needing attention. It is up to the individual to critically assess their own results and, if necessary, seek expert advice.

Publication of your CIF in IUCr journals

A basic structural check has been run on your CIF. These basic checks will be run on all CIFs submitted for publication in IUCr journals (*Acta Crystallographica*, *Journal of Applied Crystallography*, *Journal of Synchrotron Radiation*); however, if you intend to submit to *Acta Crystallographica Section C* or *E* or *IUCrData*, you should make sure that full publication checks are run on the final version of your CIF prior to submission.

Publication of your CIF in other journals

Please refer to the *Notes for Authors* of the relevant journal for any special instructions relating to CIF submission.

PLATON version of 22/08/2024; check.def file version of 21/08/2024

Datablock cu_hku_dicz_p2_0m - ellipsoid plot

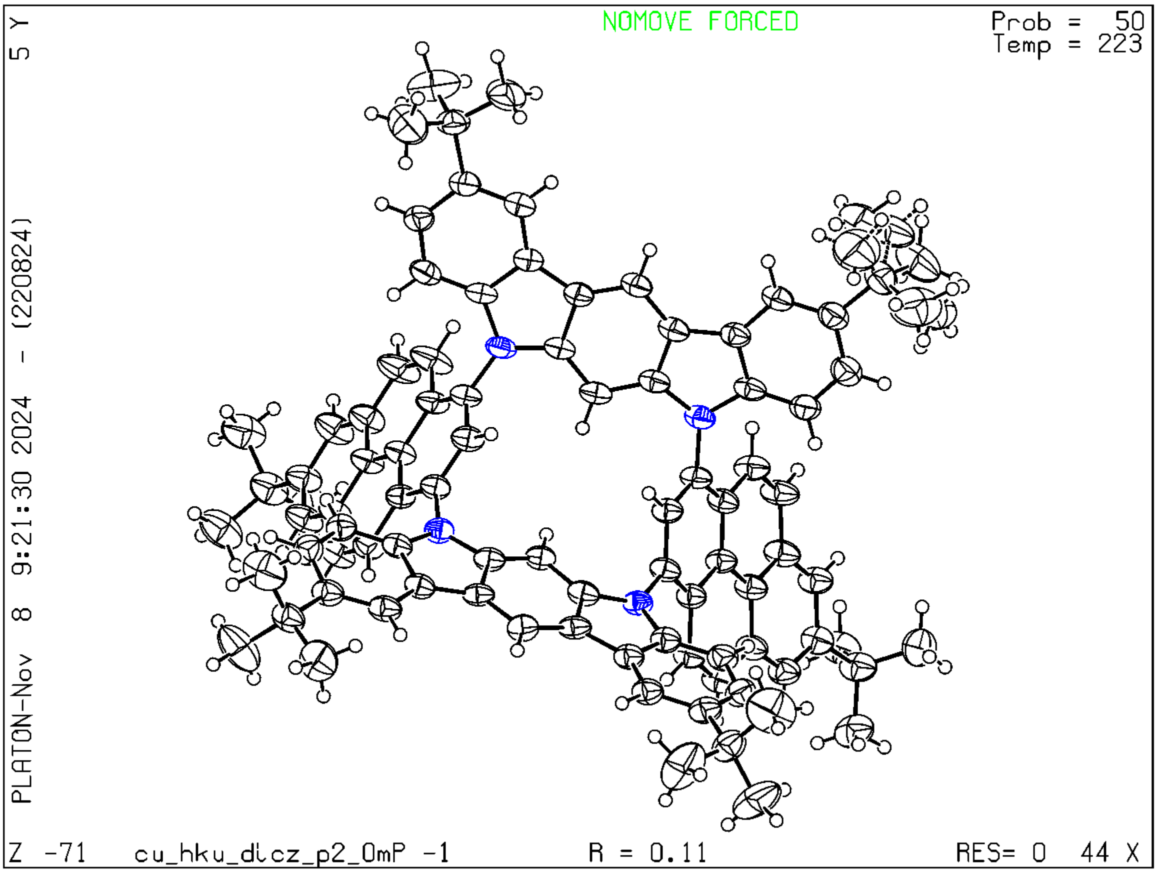

Supplement: File 2 — Crystallographic information files for compounds 3a, MC2, and MC3. [file Beilstein_J_Org_Chem-21-1917-s002.zip › MC2_cifreport.pdf]
