# Supplementary material for: Synthesis of N-doped chiral macrocycles by regioselective palladium-catalyzed arylation
Source: Beilstein J Org Chem. 2025 Sep 15;21:1917–23. doi: 10.3762/bjoc.21.149 (PMC12456077; doi:10.3762/bjoc.21.149)

checkCIF/PLATON report

Structure factors have been supplied for datablock(s) cu_n8cnb_0m_a

THIS REPORT IS FOR GUIDANCE ONLY. IF USED AS PART OF A REVIEW PROCEDURE FOR PUBLICATION, IT SHOULD NOT REPLACE THE EXPERTISE OF AN EXPERIENCED CRYSTALLOGRAPHIC REFEREE.

No syntax errors found. CIF dictionary Interpreting this report

Datablock: cu_n8cnb_0m_a

Bond precision: C-C = 0.0058 A Wavelength=1.54178

Cell:	a=14.7541(12)	b=16.6391(12)	c=17.6524(14)
	alpha=66.335(5)	beta=80.415(5)	gamma=73.726(5)
Temperature:	223 K		

	Calculated	Reported
Volume	3802.9(5)	3802.9(5)
Space group	P -1	P -1
Hall group	-P 1	-P 1
Moiety formula	C84 H44 F24 N4 [+ solvent]	C84 H44 F24 N4
Sum formula	C84 H44 F24 N4 [+ solvent]	C84 H44 F24 N4
Mr	1565.23	1565.23
Dx,g cm-3	1.367	1.367
Z	2	2
Mu (mm-1)	1.064	1.064
F000	1584.0	1584.0
F000’	1590.57	
h,k,lmax	17,20,21	17,20,21
Nref	14047	13633
Tmin,Tmax	0.871,0.890	0.653,0.753
Tmin’	0.871	

Correction method= # Reported T Limits: Tmin=0.653 Tmax=0.753
AbsCorr = MULTI-SCAN

Data completeness= 0.971 Theta(max)= 68.642

R(reflections)= 0.0748(8775) wR2(reflections)= 0.1910(13633)

S = 1.037 Npar= 1199

The following ALERTS were generated. Each ALERT has the format
test-name_ALERT_alert-type_alert-level.
Click on the hyperlinks for more details of the test.

Alert level C

```
PLAT029_ALERT_3_C _diffrn_measured_fraction_theta_full value Low .      0.975 Why?
PLAT213_ALERT_2_C Atom C2A             has ADP max/min Ratio .....        3.1 oblate
PLAT220_ALERT_2_C NonSolvent   Resd 1  C   Ueq(max)/Ueq(min) Range        3.9 Ratio
PLAT222_ALERT_3_C NonSolvent Resd 1  H   Uiso(max)/Uiso(min) Range        4.4 Ratio
PLAT234_ALERT_4_C Large Hirshfeld Difference C52      --C54      .       0.18 Ang.
PLAT242_ALERT_2_C Low    'MainMol' Ueq as Compared to Neighbors of        C23 Check
PLAT242_ALERT_2_C Low    'MainMol' Ueq as Compared to Neighbors of        C52 Check
PLAT340_ALERT_3_C Low Bond Precision on  C-C Bonds ..............    0.00583 Ang.
PLAT906_ALERT_3_C Large K Value in the Analysis of Variance ......      2.838 Check
PLAT911_ALERT_3_C Missing FCF Refl Between Thmin & STh/L=    0.600        338 Report
                8  0  0,   0  1  0,   8  1  0,   9  1  0,   3  2  0,   7  2  0,
                8  2  0,   9  2  0, -16  3  0,   7  3  0,   8  3  0,   9  3  0,
                7  4  0,   8  4  0, -15  5  0,   7  5  0,   8  5  0,   2  6  0,
                7  6  0,   9 14  0,   9 16  0,   6 17  0,   7 17  0,   8 17  0,
                9 17  0,  10 17  0,  -9-17  1,  -8-17  1,  -7-17  1,  -6-17  1,
               -7-16  1, -10-12  1, -11-11  1,  -7 -8  1,  -5 -8  1,  -2 -8  1,
               -7 -6  1,  -9 -5  1,  -8 -5  1,  -7 -5  1,  -4 -5  1,  15 -5  1,
              -13 -4  1,  -8 -4  1,  -7 -4  1,  -6 -4  1,  -8 -3  1,  -7 -3  1,
               -6 -3  1,  -2 -3  1,  16 -3  1,  -9 -2  1,  -8 -2  1,  -7 -2  1,
               -6 -2  1,  -5 -2  1,  -2 -2  1, -17 -1  1,  -9 -1  1,  -8 -1  1,
               -7 -1  1,  -6 -1  1,  -5 -1  1,  -4 -1  1,  -3 -1  1,  -2 -1  1,
               -8  0  1,  -7  0  1,  -6  0  1,  -5  0  1,  -4  0  1,  -1  0  1,
               -8  1  1,  -7  1  1,  -6  1  1,  -5  1  1,   1  1  1,   9  1  1,
              -16  2  1,   3  2  1,  16  2  1, -16  3  1,   0  3  1,   4  3  1,
                9  3  1, -15  4  1,   1  4  1,   8  4  1, -15  5  1,   8  5  1,
                8  6  1,   9  6  1,  14  6  1,   9 10  1,  15 11  1,  11 12  1,
```

Alert level G

```
PLAT002_ALERT_2_G Number of Distance or Angle Restraints on AtSite         45 Note
PLAT003_ALERT_2_G Number of Uiso or Uij Restrained non-H Atoms ...         40 Report
PLAT154_ALERT_1_G The s.u.'s on the Cell Angles are Equal ..(Note)      0.005 Degree
PLAT176_ALERT_4_G The CIF-Embedded .res File Contains SADI Records         54 Report
PLAT178_ALERT_4_G The CIF-Embedded .res File Contains SIMU Records          5 Report
PLAT187_ALERT_4_G The CIF-Embedded .res File Contains RIGU Records         10 Report
PLAT188_ALERT_3_G A Non-default SIMU Restraint Value has been used     0.0100 Report
PLAT188_ALERT_3_G A Non-default SIMU Restraint Value has been used     0.0100 Report
PLAT188_ALERT_3_G A Non-default SIMU Restraint Value has been used     0.0100 Report
PLAT188_ALERT_3_G A Non-default SIMU Restraint Value has been used     0.0100 Report
PLAT188_ALERT_3_G A Non-default SIMU Restraint Value has been used     0.0100 Report
PLAT190_ALERT_3_G A Non-default RIGU Restraint Value for First Par     0.0100 Report
PLAT190_ALERT_3_G A Non-default RIGU Restraint Value for SecondPar     0.0200 Report
PLAT190_ALERT_3_G A Non-default RIGU Restraint Value for First Par     0.0100 Report
PLAT190_ALERT_3_G A Non-default RIGU Restraint Value for SecondPar     0.0200 Report
PLAT190_ALERT_3_G A Non-default RIGU Restraint Value for First Par     0.0100 Report
PLAT190_ALERT_3_G A Non-default RIGU Restraint Value for SecondPar     0.0200 Report
PLAT190_ALERT_3_G A Non-default RIGU Restraint Value for First Par     0.0100 Report
PLAT190_ALERT_3_G A Non-default RIGU Restraint Value for SecondPar     0.0200 Report
PLAT190_ALERT_3_G A Non-default RIGU Restraint Value for First Par     0.0100 Report
PLAT190_ALERT_3_G A Non-default RIGU Restraint Value for SecondPar     0.0200 Report
```

```
PLAT190_ALERT_3_G A Non-default RIGU Restraint Value for First Par     0.0100 Report
PLAT190_ALERT_3_G A Non-default RIGU Restraint Value for SecondPar     0.0200 Report
PLAT190_ALERT_3_G A Non-default RIGU Restraint Value for First Par     0.0100 Report
PLAT190_ALERT_3_G A Non-default RIGU Restraint Value for SecondPar     0.0200 Report
PLAT190_ALERT_3_G A Non-default RIGU Restraint Value for First Par     0.0100 Report
PLAT190_ALERT_3_G A Non-default RIGU Restraint Value for SecondPar     0.0200 Report
PLAT190_ALERT_3_G A Non-default RIGU Restraint Value for First Par     0.0100 Report
PLAT190_ALERT_3_G A Non-default RIGU Restraint Value for SecondPar     0.0200 Report
PLAT190_ALERT_3_G A Non-default RIGU Restraint Value for First Par     0.0100 Report
PLAT190_ALERT_3_G A Non-default RIGU Restraint Value for SecondPar     0.0200 Report
PLAT191_ALERT_3_G A Non-default SADI Restraint Value has been used     0.0400 Report
PLAT191_ALERT_3_G A Non-default SADI Restraint Value has been used     0.0400 Report
PLAT191_ALERT_3_G A Non-default SADI Restraint Value has been used     0.0400 Report
PLAT191_ALERT_3_G A Non-default SADI Restraint Value has been used     0.0400 Report
PLAT191_ALERT_3_G A Non-default SADI Restraint Value has been used     0.0400 Report
PLAT191_ALERT_3_G A Non-default SADI Restraint Value has been used     0.0400 Report
PLAT191_ALERT_3_G A Non-default SADI Restraint Value has been used     0.0400 Report
PLAT191_ALERT_3_G A Non-default SADI Restraint Value has been used     0.0400 Report
PLAT191_ALERT_3_G A Non-default SADI Restraint Value has been used     0.0400 Report
PLAT191_ALERT_3_G A Non-default SADI Restraint Value has been used     0.0400 Report
PLAT191_ALERT_3_G A Non-default SADI Restraint Value has been used     0.0400 Report
PLAT191_ALERT_3_G A Non-default SADI Restraint Value has been used     0.0400 Report
PLAT191_ALERT_3_G A Non-default SADI Restraint Value has been used     0.0400 Report
PLAT191_ALERT_3_G A Non-default SADI Restraint Value has been used     0.0400 Report
PLAT191_ALERT_3_G A Non-default SADI Restraint Value has been used     0.0400 Report
PLAT191_ALERT_3_G A Non-default SADI Restraint Value has been used     0.0400 Report
PLAT191_ALERT_3_G A Non-default SADI Restraint Value has been used     0.0400 Report
PLAT191_ALERT_3_G A Non-default SADI Restraint Value has been used     0.0400 Report
PLAT191_ALERT_3_G A Non-default SADI Restraint Value has been used     0.0400 Report
PLAT191_ALERT_3_G A Non-default SADI Restraint Value has been used     0.0400 Report
PLAT191_ALERT_3_G A Non-default SADI Restraint Value has been used     0.0400 Report
PLAT191_ALERT_3_G A Non-default SADI Restraint Value has been used     0.0400 Report
PLAT191_ALERT_3_G A Non-default SADI Restraint Value has been used     0.0400 Report
PLAT191_ALERT_3_G A Non-default SADI Restraint Value has been used     0.0400 Report
PLAT191_ALERT_3_G A Non-default SADI Restraint Value has been used     0.0400 Report
PLAT191_ALERT_3_G A Non-default SADI Restraint Value has been used     0.0400 Report
PLAT191_ALERT_3_G A Non-default SADI Restraint Value has been used     0.0400 Report
PLAT191_ALERT_3_G A Non-default SADI Restraint Value has been used     0.0400 Report
PLAT191_ALERT_3_G A Non-default SADI Restraint Value has been used     0.0400 Report
PLAT191_ALERT_3_G A Non-default SADI Restraint Value has been used     0.0400 Report
PLAT230_ALERT_2_G Hirshfeld Test Diff for    F10      --C40      .        5.6 s.u.
PLAT230_ALERT_2_G Hirshfeld Test Diff for    F24      --C84      .        6.1 s.u.
PLAT242_ALERT_2_G Low    'MainMol' Ueq as Compared to Neighbors of        C27 Check
PLAT242_ALERT_2_G Low    'MainMol' Ueq as Compared to Neighbors of        C37 Check
PLAT242_ALERT_2_G Low    'MainMol' Ueq as Compared to Neighbors of        C66 Check
PLAT242_ALERT_2_G Low    'MainMol' Ueq as Compared to Neighbors of        C83 Check
PLAT301_ALERT_3_G Main Residue  Disorder .............(Resd    1)         18% Note
PLAT412_ALERT_2_G Short Intra XH3 .. XHn     H2A      ..H6       .       2.06 Ang.
                                                      x,y,z  =      1_555 Check
PLAT412_ALERT_2_G Short Intra XH3 .. XHn     H3C      ..H14      .       2.05 Ang.
                                                      x,y,z  =      1_555 Check
PLAT412_ALERT_2_G Short Intra XH3 .. XHn     H6       ..H2AC     .       2.06 Ang.
                                                      x,y,z  =      1_555 Check
PLAT412_ALERT_2_G Short Intra XH3 .. XHn     H14      ..H1AA     .       2.07 Ang.
                                                      x,y,z  =      1_555 Check
PLAT434_ALERT_2_G Short Inter HL..HL Contact F8       ..F22A     .       2.81 Ang.
                                                 1-x,1-y,-z  =      2_665 Check
```

```
PLAT434_ALERT_2_G Short Inter HL..HL Contact F8       ..F23      .       2.82 Ang.
                                                 1-x,1-y,-z  =      2_665 Check
PLAT606_ALERT_4_G Solvent Accessible VOID(S) in Structure ........          ! Info
PLAT720_ALERT_4_G Number of Unusual/Non-Standard Labels ..........          9 Note
              H1AA    H1AB    H1AC    H2AA    H2AB    H2AC    H3AA    H3AB
              H3AC
PLAT860_ALERT_3_G Number of Least-Squares Restraints .............        914 Note
PLAT868_ALERT_4_G ALERTS Due to the Use of _smtbx_masks Suppressed          ! Info
PLAT912_ALERT_4_G Missing # of FCF Reflections Above STh/L=  0.600         76 Note
PLAT933_ALERT_2_G Number of HKL-OMIT Records in Embedded .res File          1 Note
                1  1  1,
PLAT941_ALERT_3_G Average HKL Measurement Multiplicity ...........        2.7 Low
PLAT969_ALERT_5_G The 'Henn et al.' R-Factor-gap value ...........       2.66 Note
              Predicted wR2: Based on SigI**2  7.19 or SHELX Weight 19.28
PLAT978_ALERT_2_G Number C-C Bonds with Positive Residual Density.          0 Info
```

---

0 **ALERT level A** = Most likely a serious problem - resolve or explain
0 **ALERT level B** = A potentially serious problem, consider carefully
10 **ALERT level C** = Check. Ensure it is not caused by an omission or oversight
83 **ALERT level G** = General information/check it is not something unexpected

1 ALERT type 1 CIF construction/syntax error, inconsistent or missing data
20 ALERT type 2 Indicator that the structure model may be wrong or deficient
63 ALERT type 3 Indicator that the structure quality may be low
8 ALERT type 4 Improvement, methodology, query or suggestion
1 ALERT type 5 Informative message, check

It is advisable to attempt to resolve as many as possible of the alerts in all categories. Often the minor alerts point to easily fixed oversights, errors and omissions in your CIF or refinement strategy, so attention to these fine details can be worthwhile. In order to resolve some of the more serious problems it may be necessary to carry out additional measurements or structure refinements. However, the purpose of your study may justify the reported deviations and the more serious of these should normally be commented upon in the discussion or experimental section of a paper or in the "special_details" fields of the CIF. checkCIF was carefully designed to identify outliers and unusual parameters, but every test has its limitations and alerts that are not important in a particular case may appear. Conversely, the absence of alerts does not guarantee there are no aspects of the results needing attention. It is up to the individual to critically assess their own results and, if necessary, seek expert advice.

Publication of your CIF in IUCr journals

A basic structural check has been run on your CIF. These basic checks will be run on all CIFs submitted for publication in IUCr journals (*Acta Crystallographica*, *Journal of Applied Crystallography*, *Journal of Synchrotron Radiation*); however, if you intend to submit to *Acta Crystallographica Section C* or *E* or *IUCrData*, you should make sure that full publication checks are run on the final version of your CIF prior to submission.

Publication of your CIF in other journals

Please refer to the *Notes for Authors* of the relevant journal for any special instructions relating to CIF submission.

PLATON version of 06/01/2024; check.def file version of 05/01/2024

Datablock cu_n8cnb_0m_a - ellipsoid plot

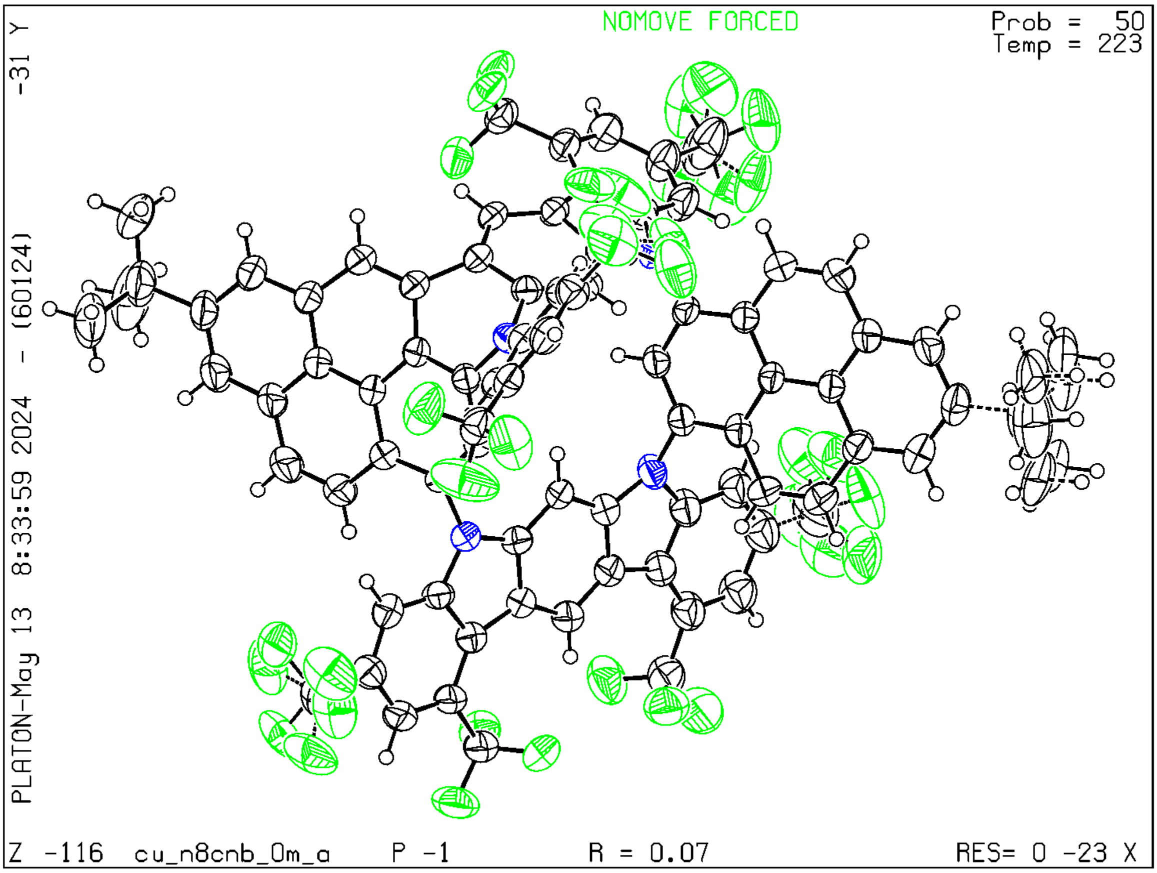

Supplement: File 2 — Crystallographic information files for compounds 3a, MC2, and MC3. [file Beilstein_J_Org_Chem-21-1917-s002.zip › MC3_cifreport.pdf]
